# Polydopamine-Coated Surfaces Promote Adhesion, Migration, Proliferation, Chemoresistance, Stemness, and Epithelial–Mesenchymal Transition of Human Prostate Cancer Cell Lines In Vitro via Integrin α_2_β_1_–FAK–JNK Signaling

**DOI:** 10.3390/ijms27020655

**Published:** 2026-01-08

**Authors:** Won Hoon Song, Ji-Eun Kim, Lata Rajbongshi, Su-Rin Lee, Yuna Kim, Seon Yeong Hwang, Sae-Ock Oh, Byoung Soo Kim, Dongjun Lee, Sik Yoon

**Affiliations:** 1 Department of Urology, Pusan National University Yangsan Hospital, Pusan National University College of Medicine, Yangsan 626-870, Republic of Korea; luchen99@hanmail.net; 2 Department of Anatomy, Pusan National University College of Medicine, Yangsan 50612, Republic of Korea; ji.eun@pusan.ac.kr (J.-E.K.); latapharm@gmail.com (L.R.); lsr020512@gmail.com (S.-R.L.); anatomy2017@pusan.ac.kr (S.Y.H.); hedgehog@pusan.ac.kr (S.-O.O.); 3 School of Medicine, Pusan National University, Yangsan 50612, Republic of Korea; yuna00kim@pusan.ac.kr; 4 School of Biomedical Convergence Engineering, Pusan National University, Yangsan 50612, Republic of Korea; bskim7@pusan.ac.kr; 5 Department of Convergence Medicine, Pusan National University College of Medicine, Yangsan 50612, Republic of Korea; lee.dongjun@pusan.ac.kr; 6 Research Institute for Convergence of Biomedical Science and Technology, Pusan National University Yangsan Hospital, Yangsan 626-870, Republic of Korea

**Keywords:** polydopamine, prostate cancer, adhesion, migration, proliferation, chemoresistance, stemness, epithelial–mesenchymal transition, FAK–JNK signaling

## Abstract

Polydopamine (PDA) surface coatings are widely used in biomedical engineering to enhance cell–substrate interactions; however, their effects on cancer-cell behavior remain unclear. In this study, we investigated how PDA-coated two-dimensional (2D) culture surfaces influence oncogenic traits of human prostate cancer (PC) cells in vitro. Using LNCaP, DU145, and PC3 cell lines, we found that PDA-coated substrates markedly increased the adhesion, migration, invasion, proliferation, and colony formation in a dose- and time-dependent manner. PDA exposure also induced epithelial–mesenchymal transition (EMT), upregulated cancer stem cell markers (*CD44*, *CD117*, *CD133*, *Sox2*, *Oct4*, and *Nanog*), and elevated expression of metastasis- and chemoresistance-associated molecules (*MMP-2*, *MMP-9*, *MDR1*, and *MRP1*). Mechanistically, PDA coatings enhanced integrin α_2_β_1_-associated cell adhesion, accompanied by increased focal adhesion kinase (FAK) phosphorylation and downstream activation of JNK signaling. Pharmacological inhibition of integrin α_2_β_1_ (BTT-3033), FAK (PF573228) and JNK (SP600125) effectively abrogated PDA-induced malignant phenotypes and restored chemosensitivity to cabazitaxel, cisplatin, docetaxel, curcumin, and enzalutamide. Collectively, these findings identify PDA-coated surfaces as a simple, efficient, and reductionist in vitro platform for studying adhesion-mediated signaling and phenotypic plasticity in PC cells, while acknowledging that further validation in three-dimensional (3D) and patient-derived models will be required to establish in vivo relevance.

## 1. Introduction

Surface modification is a cost-effective and versatile strategy that not only enhances the biocompatibility and functionality of existing biomaterials but also enables the development of novel bioactive materials with tailored surface properties [1]. Among recent advances, bioinspired polyphenol-based coatings have attracted significant attention due to their ability to form multifunctional conformal films on a wide range of substrates [2]. Polydopamine (PDA), a synthetic polyphenol inspired by mussel adhesive proteins, has emerged as a highly stable coating material because of its strong universal adhesion and abundant reactive functional groups [3,4]. PDA contains both catechol and amine moieties that confer exceptional interfacial binding to virtually all types of inorganic and organic surfaces, regardless of their chemical composition [5]. Since its discovery, PDA has been recognized for its unique physicochemical versatility, making it a promising material for applications in materials science, nanotechnology, regenerative medicine, and theranostics [6]. Owing to these attributes, PDA has rapidly become one of the most widely adopted surface modification agents over the past decade [7]. Numerous studies have demonstrated that PDA coatings and their derivatives improve the surface functionality of diverse substrates, including gold nanoparticles [8], polymeric nanocarriers [9], bone substitutes [10], synthetic polymer scaffolds [11], and biomedical implants [12,13].

The extracellular matrix (ECM) is a complex three-dimensional (3D) network of multidomain macromolecules that provides structural support; regulates essential cellular behaviors, including adhesion, proliferation, migration, polarity, differentiation, and apoptosis; and serves as a reservoir for signaling molecules that orchestrate numerous biological processes [14,15,16]. Cell adhesion is essential for maintaining tissue integrity and enabling cells to interact with their environment via specific molecular mechanisms [17]. It underlies both cell–cell and cell–ECM interactions, supporting the survival of anchorage-dependent cells and initiating signaling cascades that control cell proliferation, migration, and differentiation [18]. In addition to structural support, cell adhesion functions as a critical regulatory mechanism that influences cell renewal, communication, embryonic morphogenesis, angiogenesis, and tissue homeostasis [18,19]. These interactions are mediated by cell adhesion molecules (CAMs), a diverse group of surface proteins that anchor cells to neighboring cells or the ECM, and act as bidirectional signaling mediators [19]. Among the CAMs, integrins are the most prominent family of transmembrane receptors. Integrins are composed of α and β subunits that form heterodimers. They physically link the ECM to the cytoskeleton and serve as key transducers of biochemical and mechanical cues. Through these dual signaling roles, integrins regulate a broad range of biological processes, including cell adhesion, proliferation, migration, apoptosis, inflammation, angiogenesis, and tissue repair in both physiological and pathological contexts [20,21].

Numerous studies have demonstrated that PDA coatings promote cellular adhesion across a broad spectrum of cell types, including fibroblasts, endothelial cells, osteoblasts, neural cells, epithelial cells, and stem cells [22,23,24,25]. In addition to enhancing adhesion, PDA coatings stimulate the proliferation of multiple cell populations—such as endothelial cells, osteoblasts, stem cells, fibroblasts, and keratinocytes—and facilitate the migration of mesenchymal stem cells and Schwann cells [23,26,27,28,29,30]. Furthermore, PDA-coated surfaces enhance the cellular colonization of biomaterial implants, demonstrating their ability to modulate host–material interactions and biological responses to prosthetic materials [12]. Collectively, these findings highlight the bioactive potential of PDA as a multifunctional coating that supports cell adhesion, proliferation, and migration, thereby contributing to wound healing and tissue regeneration.

Despite substantial progress in elucidating the beneficial biological effects of PDA, its influence on malignant cells and the underlying molecular mechanisms remain poorly understood. Although several studies have investigated PDA in the context of nanoparticle-based cancer therapy [31], biological and mechanistic insights into how PDA influences tumor-cell behavior and signaling remain limited. A deeper understanding of these activities and their underlying mechanisms is crucial for elucidating the potential role of PDA in tumor progression and advancing its application in cancer theranostics.

Given that PDA coatings robustly enhance cell–substrate adhesion across multiple cell types [3,32,33], we reasoned that PDA-induced phenotypic changes in prostate cancer (PC) cells would be mediated primarily through integrin-dependent signaling pathways. Among integrin-associated signaling molecules, focal adhesion kinase (FAK) plays a pivotal role in transducing mechanical and biochemical cues from the extracellular environment to regulate cytoskeletal remodeling and key cellular processes, including adhesion, migration, proliferation, survival, epithelial–mesenchymal transition (EMT), and therapy resistance [32,34,35,36,37,38,39,40,41]. Dysregulation of FAK signaling has been widely implicated in tumor initiation, invasion, metastasis, and chemoresistance, largely through the promotion of EMT and acquisition of stem-like traits [40,42,43,44,45,46].

Importantly, FAK activation engages downstream mitogen-activated protein kinase pathways, including the c-Jun N-terminal kinase (JNK) pathway, which plays a critical role in EMT induction, cancer stemness, invasion, and chemoresistance in PC and other solid tumors [33,47,48,49,50]. Given that human PC frequently progresses to an aggressive, therapy-resistant disease characterized by enhanced migratory capacity, stemness, and EMT features [51,52], these observations provide a strong mechanistic rationale for focusing on the integrin–FAK–JNK signaling axis as a key mediator of PDA-induced malignant phenotypic plasticity in PC cells.

In this study, we systematically examined how PDA-coated two-dimensional (2D) culture surfaces influence key oncogenic traits of human PC cells using a comprehensive panel of cellular, molecular, and pharmacological assays. Specifically, we assessed cell adhesion, migration, invasion, proliferation, clonogenic growth, EMT, cancer stem cell (CSC) marker expression, and chemoresistance following exposure to PDA-coated substrates. Mechanistic investigations focused on integrin-dependent signaling, with particular emphasis on integrin α_2_β_1_-mediated activation of FAK and its downstream JNK signaling. The functional relevance of this pathway was interrogated using pharmacological inhibitors targeting integrin α_2_β_1_, FAK, and JNK. Together, these approaches were designed to define how surface-derived adhesive cues alone can modulate PC cell plasticity and aggressive behavior.

To ensure biological relevance across clinically distinct PC states, we employed three widely used human PC cell lines—LNCaP, DU145, and PC3—which together represent major stages of PC progression [53,54,55]. LNCaP cells are androgen receptor (AR)-positive and model androgen-dependent disease, whereas DU145 and PC3 cells are AR-negative and represent more advanced, androgen-independent and metastatic phenotypes [53,56,57,58,59,60,61]. PC3 cells, in particular, are derived from bone metastases and exhibit highly aggressive and therapy-resistant behavior [53,60,62]. The use of these complementary cell lines allowed us to determine whether PDA-induced adhesion signaling and downstream phenotypic changes are conserved across hormonally sensitive and castration-resistant PC contexts, thereby strengthening the generalizability of our findings.

Therefore, this study aimed to systematically examine the effects of PDA-coated 2D surfaces on key oncogenic traits of human PC cells, including adhesion, migration, invasion, proliferation, chemoresistance, stemness, and EMT. Based on the established role of integrin-mediated adhesion signaling and the integrin–FAK–JNK axis in PC progression, we hypothesized that PDA-enhanced cell–substrate adhesion selectively activates integrin-dependent FAK and downstream JNK signaling, thereby promoting malignant phenotypic plasticity in PC cells. This hypothesis provided the mechanistic framework for the pathway-focused analyses performed in the present study and for defining how surface-derived adhesive cues alone can modulate PC cell behavior in vitro.

## 2. Results

### 2.1. PDA Coating Enhances the Adhesion of Human PC Cells

**Figure 1:**
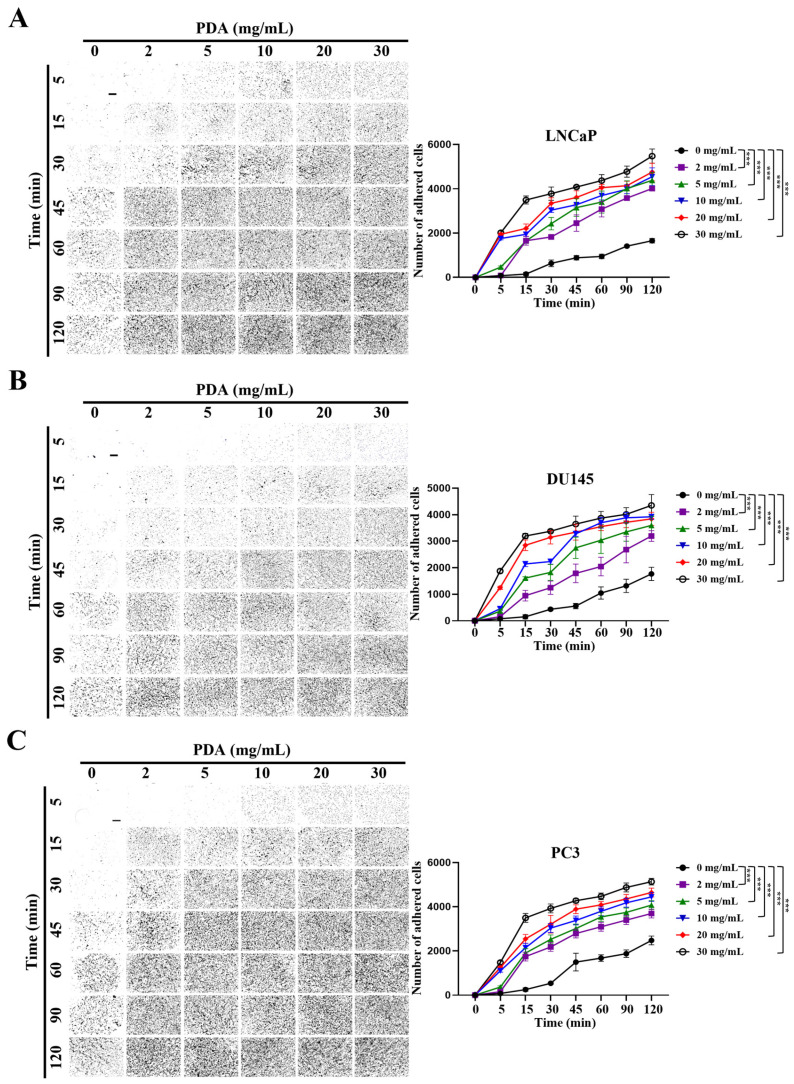
PDA coating enhances the adhesion of human PC cells. Representative phase-contrast images and quantification of crystal-violet-stained LNCaP (**A**), DU145 (**B**), and PC3 (**C**) cells seeded on polystyrene surfaces coated with increasing PDA concentrations (0–30 mg/mL) and assessed at 5–120 min (original magnification ×40). PDA coating significantly enhanced cell adhesion in a concentration- and time-dependent manner across all of the cell lines. Graphs depict the interaction between PDA concentration and incubation time on relative cell adhesion. Data are presented as mean ± SD (*n* = 3). Statistical significance compared with the control (0 mg/mL PDA) at the same time point was determined using Bonferroni’s post hoc test following two-way ANOVA and is indicated as *** *p* < 0.001. Scale bar = 650 µm.

To evaluate the effect of PDA coating on PC cell adhesion, polystyrene plates were coated with increasing concentrations of PDA (0–30 mg/mL), and the attachment of LNCaP, DU145, and PC3 cells was quantified at multiple time points using crystal violet staining (Figure 1). Across all three cell lines, the PDA coating significantly enhanced cell adhesion in a concentration- and time-dependent manner (Figure 1).

For LNCaP cells, two-way ANOVA revealed significant main effects of PDA concentration and incubation time, as well as a significant concentration × time interaction (Figure 1A), indicating that PDA-mediated adhesion depended on both variables. At the early 5 min time point, PDA concentrations ≥ 5 mg/mL induced a marked increase in cell attachment relative to uncoated controls (*p* < 0.001), whereas 2 mg/mL PDA did not yet produce a statistically significant effect. With increasing incubation time (15–120 min), adhesion increased substantially across all PDA concentrations, and all PDA-coated surfaces (≥2 mg/mL) exhibited significantly higher cell attachment than controls (*p* < 0.001). Higher PDA concentrations (10–30 mg/mL) promoted more rapid and robust adhesion, particularly during early incubation periods, whereas cells on uncoated surfaces showed minimal attachment at early time points (Figure 1A).

DU145 cells exhibited a similar response pattern (Figure 1B). Two-way ANOVA demonstrated significant effects of PDA concentration, incubation time, and their interaction (all *p* < 0.001). At 5 min, significant enhancement of adhesion was observed at PDA concentrations ≥ 5 mg/mL, while at later time points (15–120 min), all PDA concentrations ≥ 2 mg/mL significantly increased cell attachment compared with uncoated controls (*p* < 0.001).

PC3 cells showed the most pronounced adhesion response to PDA coating (Figure 1C). Two-way ANOVA again revealed significant effects of PDA concentration, incubation time, and their interaction (all *p* < 0.001). Similarly to the other cell lines, PDA concentrations ≥ 5 mg/mL significantly enhanced adhesion at 5 min, whereas all concentrations ≥ 2 mg/mL produced robust increases in attachment at 15–120 min relative to uncoated controls (*p* < 0.001).

Taken together, PDA-mediated adhesion increased in a concentration- and time-dependent manner across all three PC cell lines. A PDA concentration of 10 mg/mL consistently produced strong, rapid, and reproducible enhancement of cell attachment at both early and later time points, whereas further increases in PDA concentration (20–30 mg/mL) resulted in only modest additional gains, indicating the onset of a saturation effect. Based on its robust efficacy, consistency across cell lines, and minimal incremental benefit at higher concentrations, 10 mg/mL PDA was selected as the optimal coating concentration for subsequent adhesion-dependent functional assays.

Overall, these results demonstrate that PDA coating markedly accelerates the early attachment of human PC cells, with the magnitude of enhancement varying among cell lines.

### 2.2. PDA Coating Promotes the Migration of Human PC Cells

**Figure 2:**
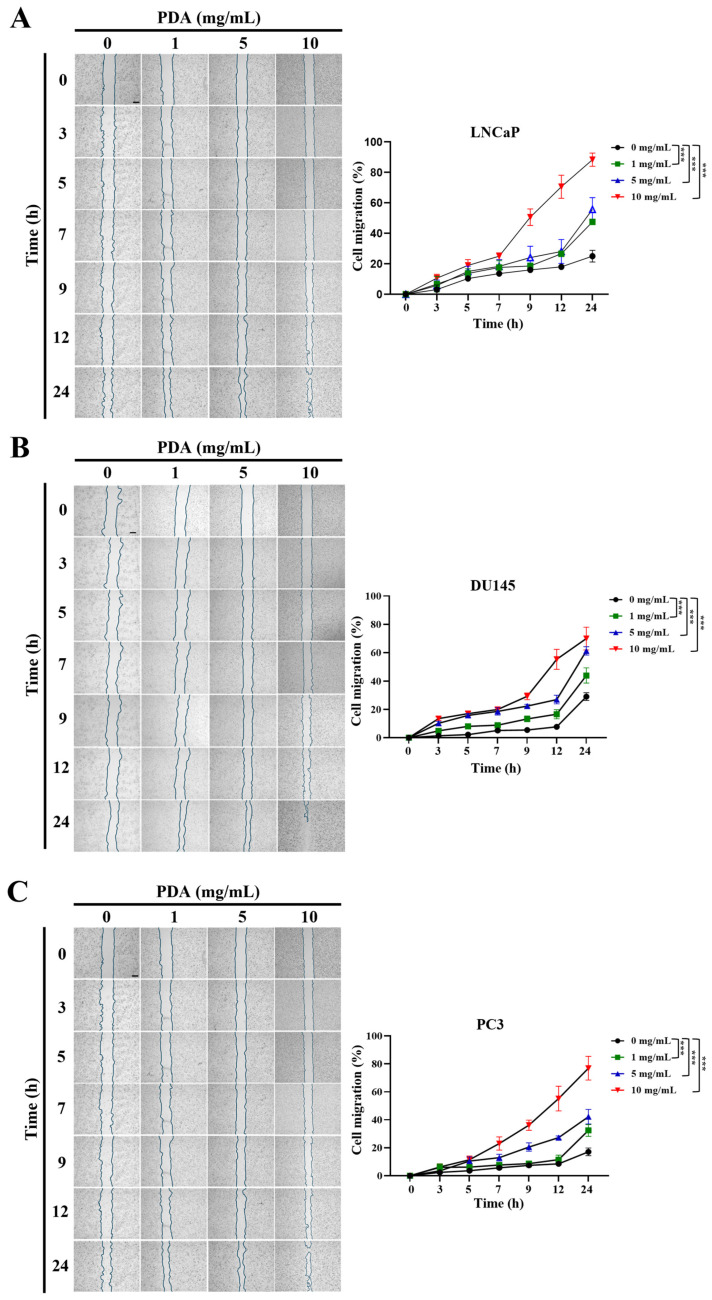
PDA coating enhances the migration of human PC cells. Scratch-wound assays showing the migration of LNCaP (**A**), DU145 (**B**), and PC3 (**C**) cells seeded on polystyrene surfaces coated with increasing concentrations of PDA (0–10 mg/mL). Wound closure was monitored over 0–24 h using phase-contrast microscopy (original magnification ×40). PDA coating significantly accelerated cell migration in a concentration- and time-dependent manner across all of the cell lines. Graphs depict the interaction between PDA concentration and incubation time on relative wound closure. Data are presented as mean ± SD (*n* = 3). Statistical significance compared with the control (0 mg/mL PDA) at the same time point was determined using Bonferroni’s post hoc test following two-way ANOVA and is indicated as *** *p* < 0.001. Scale bar = 650 µm.

To assess the effect of PDA coating on PC cell migration, polystyrene plates were coated with increasing PDA concentrations (0–10 mg/mL), and cell motility was evaluated using a scratch-wound assay over a 24 h period (Figure 2).

For LNCaP cells, two-way ANOVA revealed significant main effects of PDA concentration and incubation time, as well as a significant concentration × time interaction (all *p* < 0.001), indicating that PDA-mediated migration depended on both variables (Figure 2A). Post hoc analysis demonstrated that PDA coating significantly accelerated wound closure in a concentration- and time-dependent manner. Notably, 10 mg/mL PDA induced the most pronounced migratory response at early and intermediate time points, whereas lower concentrations produced weaker or delayed effects. By 24 h, all PDA concentrations significantly enhanced migration relative to uncoated controls (Figure 2A).

DU145 cells exhibited a similar response pattern (Figure 2B). Two-way ANOVA again identified significant effects of PDA concentration, incubation time, and their interaction (all *p* < 0.001). PDA coating significantly promoted DU145 cell migration in a time- and concentration-dependent manner, with 10 mg/mL PDA consistently eliciting the strongest migratory response during early and intermediate phases of the assay (Figure 2B). At 24 h, migration was significantly increased at all PDA concentrations compared with uncoated controls (Figure 2B).

PC3 cells also showed robust enhancement of migration in response to PDA coating (Figure 2C). Two-way ANOVA confirmed significant effects of PDA concentration, incubation time, and their interaction (all *p* < 0.001). As observed for LNCaP and DU145 cells, 10 mg/mL PDA produced the most pronounced acceleration of wound closure at early and intermediate time points, whereas lower concentrations exhibited less marked effects (Figure 2C). By 24 h, all PDA concentrations significantly increased PC3 cell migration relative to controls (Figure 2C).

Collectively, these results demonstrated that PDA coating promotes the migration of human PC cells in a concentration- and time-dependent manner, with the magnitude of the migratory response varying among cell lines.

### 2.3. PDA Coating Enhances the Invasion of Human PC Cells

**Figure 3:**
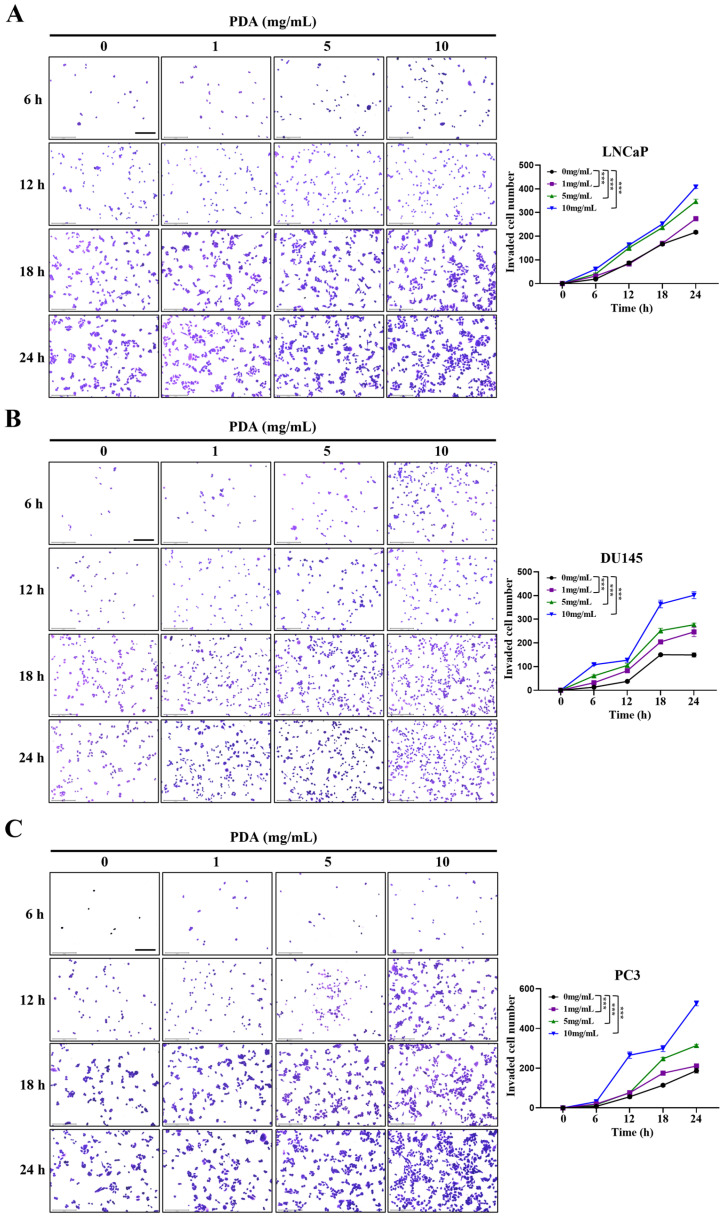
PDA coating enhances the invasive behavior of human PC cells. Representative images of the hydrogel invasion assay showing LNCaP (**A**), DU145 (**B**), and PC3 (**C**) cells cultured on polystyrene surfaces coated with increasing concentrations of PDA. PDA-coated plates significantly increased the invasive capacity of all three cell lines compared with uncoated control surfaces (original magnification ×100). Graphs depict the interaction between PDA concentration and incubation time on relative invasion. Data are presented as mean ± SD (*n* = 3). Statistical significance compared with the control (0 mg/mL PDA) at the same time point was determined using Bonferroni’s post hoc test following two-way ANOVA and is indicated as *** *p* < 0.001. Scale bars: 275 μm.

To evaluate the effect of PDA coating on PC cell invasion, polystyrene surfaces were coated with increasing concentrations of PDA (0–10 mg/mL), and invasive capacity was assessed over 24 h period using hydrogel-based invasion assays (Figure 3A–C).

For LNCaP cells, two-way ANOVA revealed significant main effects of PDA concentration and incubation time, as well as a significant concentration × time interaction (all *p* < 0.001), indicating that PDA-mediated invasion depended on both variables (Figure 3A). Post hoc analysis demonstrated that PDA coating significantly enhanced invasion in a concentration- and time-dependent manner. At early time points, significant increases were primarily observed at higher PDA concentrations (5–10 mg/mL), whereas at later time points (18–24 h), all PDA concentrations significantly increased invasion relative to uncoated controls (Figure 3A). Notably, 10 mg/mL PDA consistently induced the most pronounced invasive response across all incubation times (Figure 3A).

DU145 cells exhibited a similar invasion profile (Figure 3B). Two-way ANOVA again identified significant effects of PDA concentration, incubation time, and their interaction (all *p* < 0.001). PDA coating significantly promoted DU145 cell invasion in a time- and concentration-dependent manner, with higher PDA concentrations required to elicit significant effects at early time points (Figure 3B). At later time points, invasion was significantly increased at all PDA concentrations, with 10 mg/mL PDA consistently producing the strongest response (Figure 3B).

PC3 cells showed robust enhancement of invasive behavior in response to PDA coating (Figure 3C). Two-way ANOVA confirmed significant effects of PDA concentration, incubation time, and their interaction (all *p* < 0.001). Significant increases in invasion at early time points were predominantly observed at 10 mg/mL PDA, whereas at later time points all PDA concentrations significantly enhanced invasion compared with uncoated controls. As observed in the other cell lines, 10 mg/mL PDA consistently induced the most pronounced invasive response across all time points (Figure 3C).

Collectively, these findings demonstrate that a PDA coating promotes invasive behavior across human PC cell lines in a concentration- and time-dependent manner compared with uncoated control surfaces.

### 2.4. PDA Coating Stimulates the Proliferation of Human PC Cells

In contrast to assays assessing cell migration, invasion, EMT- and stemness-associated gene expression, and chemoresistance, preliminary dose–response analyses revealed that a lower PDA concentration (1 mg/mL) was sufficient to elicit a robust proliferative response. Accordingly, this concentration was used for proliferation assays to avoid saturation-related confounding effects and to enable accurate quantification of changes in cell growth.

**Figure 4:**
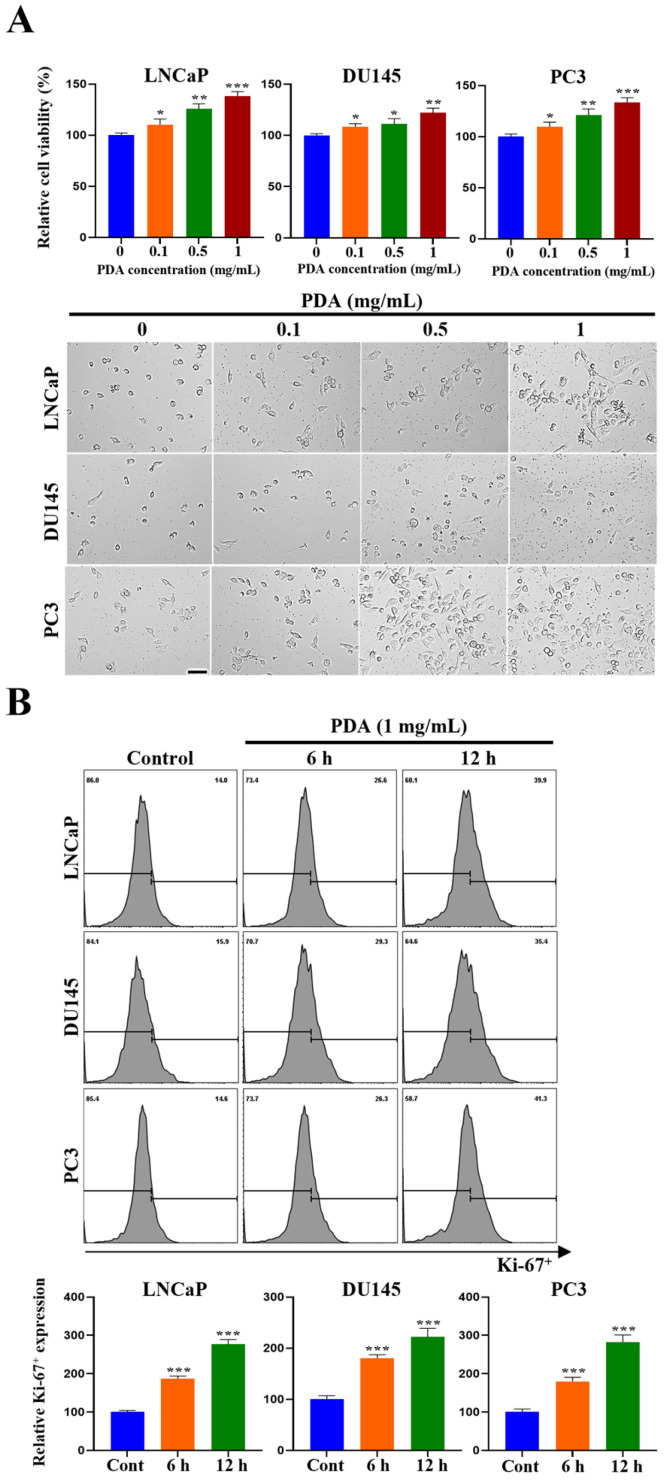
PDA coating enhances the proliferation of human PC cells. (**A**) Phase-contrast images and results of CCK-8 assays of LNCaP, DU145, and PC3 cells cultured for 24 h on polystyrene surfaces coated with increasing PDA concentrations (0–1 mg/mL) (original magnification ×100). The PDA coating resulted in a dose-dependent increase in cell proliferation. (**B**) Flow cytometry analysis results for intracellular Ki-67 expression in LNCaP, DU145, and PC3 cells seeded on 1 mg/mL PDA-coated surfaces, showing a time-dependent increase in proliferative activity (data are presented as mean ± SD; * *p* < 0.05, ** *p* < 0.01, and *** *p* < 0.001 vs. uncoated control; scale bar = 275 µm).

The proliferation of LNCaP, DU145, and PC3 cells was evaluated using a CCK-8 assay after incubation on polystyrene surfaces coated with increasing PDA concentrations (0–1 mg/mL) (Figure 4A). The PDA coating significantly increased cell proliferation at 24 h in all three cell lines, with the greatest effect observed at 1 mg/mL (Figure 4A). Lower PDA concentrations (0.1–0.5 mg/mL) also produced moderate increases. Consistent with these findings, phase-contrast images showed higher cell densities on the PDA-coated surfaces (Figure 4A).

To further assess the proliferative activity, Ki-67 levels were analyzed by flow cytometry following adhesion to 1 mg/mL PDA-coated surfaces (Figure 4B). All three cell lines exhibited a time-dependent increase in Ki-67 expression between 6 and 12 h compared with the uncoated controls.

Collectively, these results indicated that a PDA coating enhances the proliferative capacity of human PC cells in a dose- and time-dependent manner.

### 2.5. PDA Coating Enhances Integrin α_2_β_1_ Expression and FAK Phosphorylation in Human PC Cells

**Figure 5:**
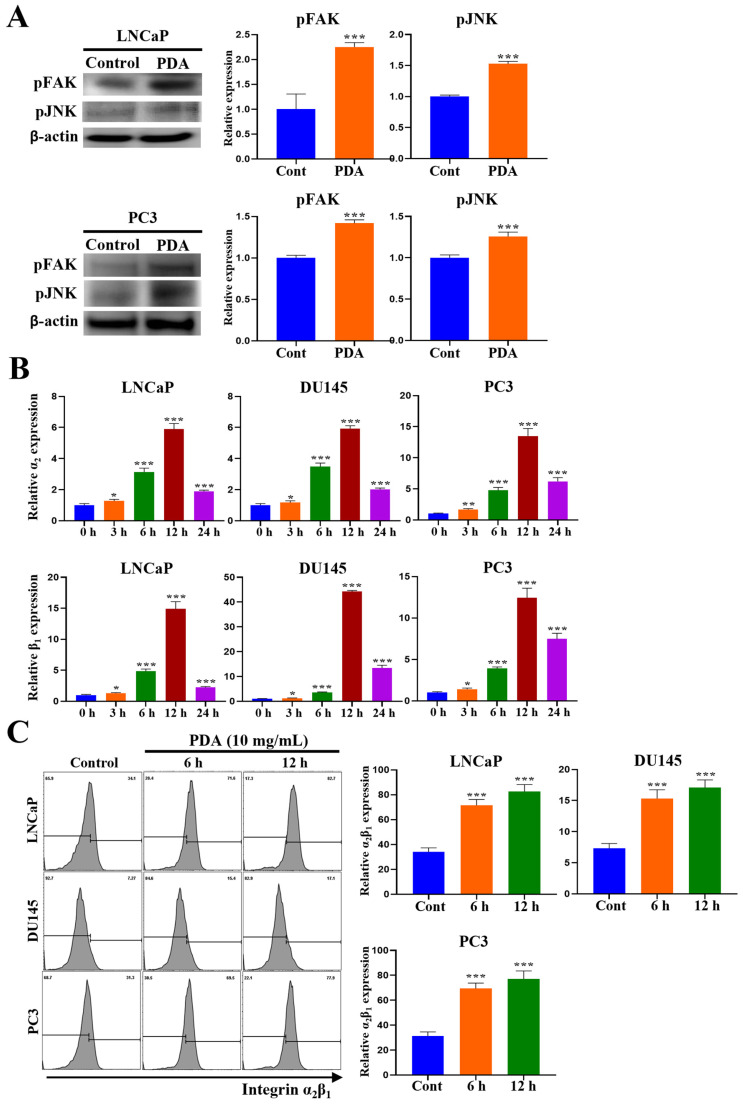
PDA coating enhances FAK phosphorylation and α_2_β_1_ integrin expression in human PC cells. (**A**) Western blot analysis results for phosphorylated FAK (Tyr576/577) in LNCaP and PC3 cells following adhesion to 10 mg/mL PDA-coated polystyrene surfaces for 30 min. (**B**) qRT-PCR analysis results for integrin subunit mRNA expression (*α*_2_, *α*_3_, *α*_5_, *α_v_*, *β*_1_, *β*_3_, and *β*_8_) in LNCaP, DU145, and PC3 cells after adhesion to 10 mg/mL PDA-coated surfaces for 3–24 h. Early transcriptional peaks (3–12 h) followed by partial attenuation at 24 h reflect dynamic adhesion signaling during cell spreading and stabilization. (**C**) Flow cytometry analysis results for surface α_2_β_1_ expression in LNCaP, DU145, and PC3 cells following adhesion to PDA-coated surfaces for 6 and 12 h. The PDA coating induced a time-dependent increase in FAK phosphorylation and α_2_β_1_ expression across all cell lines (data are presented as mean ± SD; * *p* < 0.05, ** *p* < 0.01, and *** *p* < 0.001 vs. uncoated control).

Phosphorylated FAK (pFAK) was used as an indicator of integrin signaling activation [63,64,65] (Figure 5A). To assess whether the PDA coating induced FAK activation, the pFAK levels in LNCaP and PC3 cells were measured following adhesion to PDA-coated surfaces. Western blot analyses using anti-phospho-FAK (Tyr576/577) antibody revealed significantly increased pFAK levels in both cell lines after adhesion to 10 mg/mL PDA for 30 min, with a 2.3-fold increase in LNCaP cells (*p* < 0.01) and 4.2-fold increase in PC3 cells (*p* < 0.001) compared to uncoated controls (Figure 5A).

To determine whether PDA-induced adhesion altered integrin expression, a qRT-PCR was performed for the integrin subunits associated with PC progression (*α*_2_, *α*_3_, *α*_5_, *α_v_*, *β*_1_, *β*_3_, and *β*_8_) [66,67,68,69]. The adhesion of PC3 cells to 10 mg/mL PDA-coated surfaces for 3, 6, and 12 h significantly increased *integrin α*_2_ expression by 1.7-(*p* < 0.01), 4.8-(*p* < 0.001), and 13.5-fold (*p* < 0.001), respectively, compared with uncoated controls (Figure 5B). The LNCaP and DU145 cells showed similar increases at 6 and 12 h, with 3.1- and 5.9-fold upregulation values in LNCaP cells and 3.5- and 5.9-fold increases in DU145 cells, respectively (*p* < 0.001 for all time points) (Figure 5B). *Integrin β*_1_ expression was also markedly upregulated following adhesion to PDA-coated surfaces (Figure 5B). In the PC3 cells, *β*_1_ expression increased by 3.9-fold (*p* < 0.001) and 12.4-fold (*p* < 0.001) at 6 and 12 h, respectively. The LNCaP cells exhibited 4.8- and 14.9-fold increases, whereas the DU145 cells showed 3.6- and 44.2-fold increases, respectively, under identical conditions (*p* < 0.001 for all time points). At 24 h, the expression levels of *integrin α*_2_ and *β*_1_ showed partial attenuation compared with peak levels at 12 h, while remaining significantly elevated relative to uncoated control surfaces (Figure 5B), consistent with the dynamic regulation of adhesion-associated gene expression following initial integrin engagement.

Flow cytometry confirmed a time-dependent increase in surface α_2_β_1_ integrin expression across all cell lines after adhesion to 10 mg/mL PDA-coated surfaces (Figure 5C). In the PC3 cells, the α_2_β_1_ levels increased by 2.2-fold (*p* < 0.001) at 6 h and 2.5-fold (*p* < 0.001) at 12 h, with comparable increases observed in LNCaP (2.1- to 2.4-fold) and DU145 cells (2.1- to 2.3-fold) (*p* < 0.001 for all measurements). Collectively, these findings demonstrated that the PDA coating enhanced α_2_β_1_ integrin expression and FAK phosphorylation in a time-dependent manner across human PC cell lines (Figure 5).

### 2.6. PDA Coating Upregulates the Expression of Stem Cell and EMT-Associated Markers in Human PC Cells

**Figure 6:**
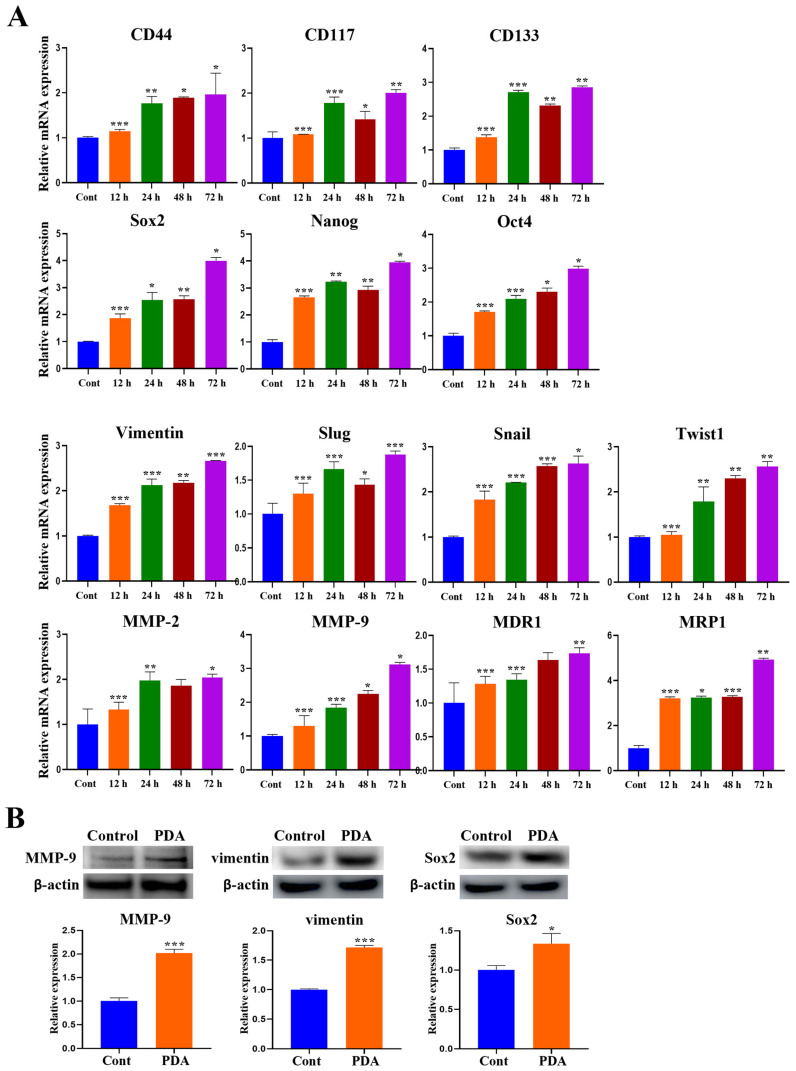
PDA coating upregulates stemness- and EMT-associated markers in human PC cells. (**A**) qRT-PCR analysis results for CSC-related genes (*CD44*, *CD117*, *CD133*, *SOX2*, *OCT4*, *NANOG*) and EMT-associated markers (*vimentin*, *SLUG*, *SNAIL*, *TWIST1*, *MMP-2*, *MMP-9*, *MDR1*, *MRP1*) in DU145 cells following adhesion to 10 mg/mL PDA-coated polystyrene surfaces for 12–72 h. The PDA coating induced a time-dependent increase in the expression of stemness- and EMT-related genes. (**B**) Western blot analysis results for MMP-9, vimentin, and SOX2 protein levels in LNCaP cells after 6 and 12 h of adhesion to PDA-coated surfaces (data are presented as mean ± SD; * *p* < 0.05, ** *p* < 0.01, and *** *p* < 0.001 vs. uncoated control).

To determine whether a PDA coating influences cancer stemness, a qRT-PCR analysis was performed for cancer stem cell (CSC)-associated genes in human DU145 cells (Figure 6A). PDA-coated surfaces significantly increased the expression of *CD44*, *CD117*, and *CD133*, as well as that of pluripotency-related transcription factors, including sex-determining region Y-box 2 (*Sox2*), octamer-binding transcription factor-4 (*Oct4*), and homeobox protein NANOG transcription factor (*Nanog*) (Figure 6A). Additionally, a qRT-PCR analysis was performed to investigate the molecular mechanisms underlying the PDA-mediated regulation of malignant cell behavior and tumor progression. The PDA coating also upregulated key regulators of EMT, including *Slug*, *Snail*, and *Twist1* (pivotal EMT-associated transcription factors); *vimentin* (a key EMT marker); and matrix metalloproteinase-2 (*MMP-2*) and *MMP-9* (crucial EMT-associated enzymes), along with the multidrug resistance-related genes *MDR1* and *MRP1* (critical proteins associated with the EMT process) (Figure 6A). These effects were time-dependent. PDA coating progressively increased the expression of CSC-, EMT-, and MMP-associated genes over 12–72 h (Figure 6A). Similar patterns were observed in PC3 cells, with the expression profiles confirmed in PC3 cells by extended analysis (Figure S1).

Western blotting further demonstrated the PDA-induced elevation of MMP-9, vimentin, and Sox2 protein levels, with 1.3-, 1.7-, and 1.3-fold increases in LNCaP cells at 72 h, respectively (*p* < 0.05–0.001) (Figure 6B), and a two-fold increase in MMP-9 in PC3 cells at 72 h.

Collectively, these findings indicated that a PDA coating promotes cancer stemness, EMT activation, matrix remodeling, and drug-resistance-associated gene expression in human PC cells.

### 2.7. FAK Activation Mediates PDA-Induced Adhesion, Migration, and Invasion of Human PC Cells

**Figure 7:**
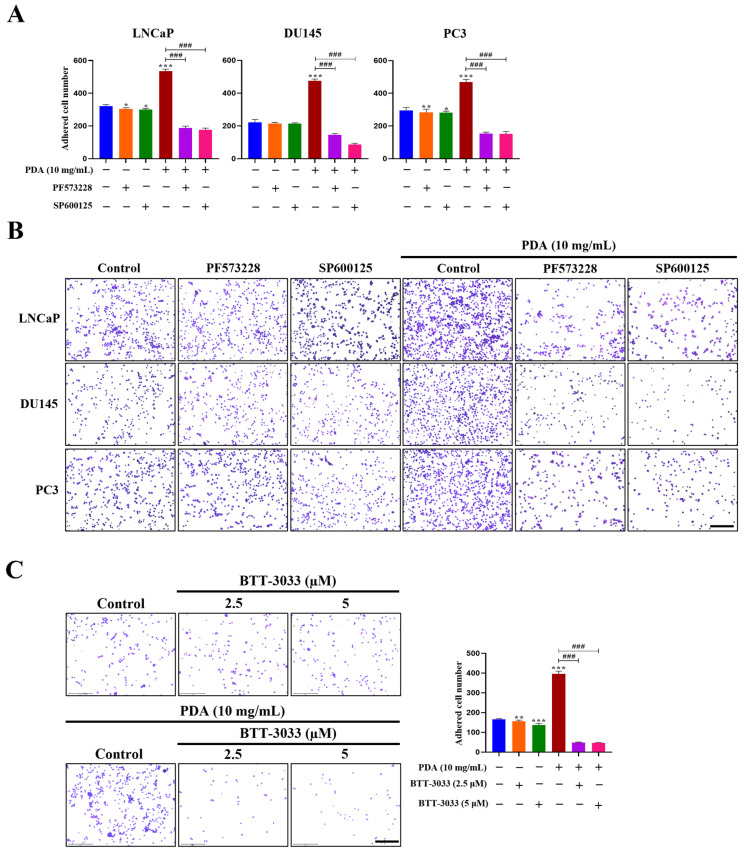
Effects of FAK, JNK, and integrin α_2_β_1_ inhibition on PDA-induced adhesion of human PC cells. (**A**) Quantification of LNCaP, DU145. and PC3 cell adhesion to 10 mg/mL PDA-coated polystyrene surfaces, assessed by crystal violet staining. Cells were pretreated with the FAK inhibitor PF573228 (10 µM) or JNK inhibitor SP600125 (30 µM) prior to adhesion. (**B**) Representative images of LNCaP, DU145. and PC3 cells following adhesion to PDA-coated surfaces in the presence or absence of PF573228 or SP600125 (original magnification ×100). (**C**) Representative images of PC3 cells following adhesion PDA-coated surfaces in the presence or absence of the integrin α_2_β_1_ inhibitor BTT-3033 (2.5 and 5 µM) (original magnification ×100), along with quantification of PC3 cell adhesion assessed by crystal violet staining. Cells were pretreated with BTT-3033 prior to adhesion. Data are expressed as relative values normalized to uncoated control surfaces and are presented as mean ± SD from three independent experiments. * *p* < 0.05, ** *p* < 0.01, and *** *p* < 0.001 versus uncoated control; ^###^ *p* < 0.001 versus PDA-treated group. Scale bar = 275 µm.

To determine whether FAK mediates PDA-induced adhesion, migration, and invasion of human PC cells, pharmacological inhibition studies were performed. PC3, LNCaP, and DU145 cells showed markedly increased adhesion to 10 mg/mL PDA-coated surfaces compared to uncoated controls, showing 22.8-, 22.9-, and 26.7-fold increases, respectively, compared with uncoated controls (*p* < 0.01–0.001; Figure 7). Pretreatment with the FAK inhibitor PF573228 (10 μM), JNK inhibitor SP600125 (30 μM), or integrin α_2_β_1_ inhibitor BTT-3033 (2.5 and 5 μM) effectively abolished the PDA-induced adhesion in all cell lines (Figure 7). Notably, treatment with PF573228, SP600125, and BTT-3033 exerted minimal effects on basal adhesion in cells cultured on uncoated control surfaces (Figure 7C). These results indicate that integrin–FAK–JNK signaling is selectively amplified in the context of enhanced cell adhesion under PDA-coated conditions, rather than reflecting nonspecific inhibitory effects of the compounds.

**Figure 8:**
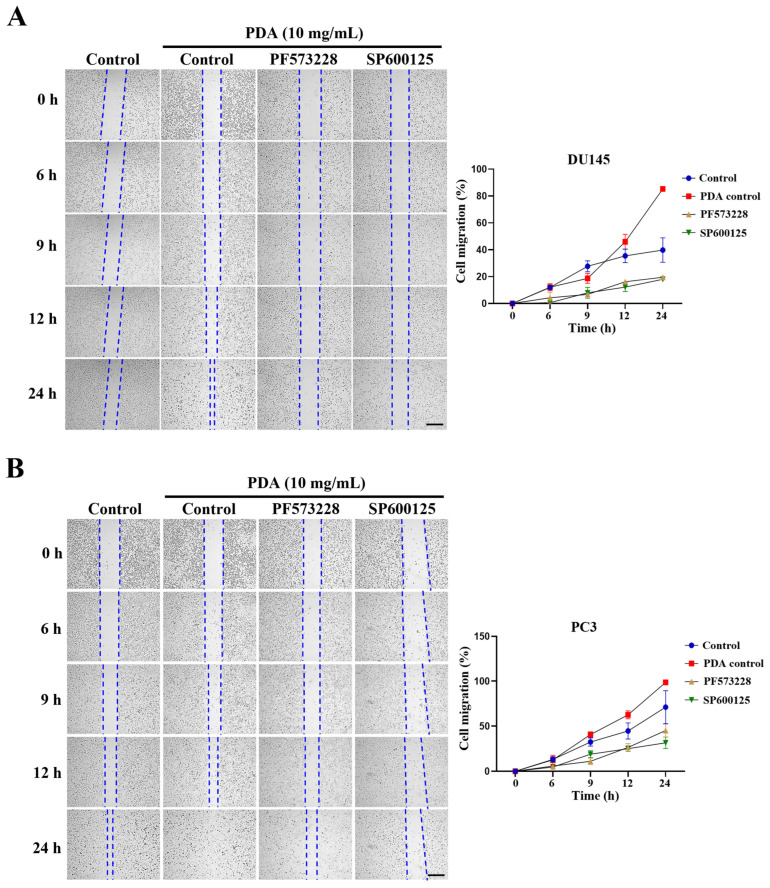
PDA coating enhances migration of human PC cells, which is suppressed by FAK or JNK inhibition. (**A**,**B**) Representative phase-contrast images from scratch-wound assays of DU145 (**A**) and PC3 (**B**) cells cultured on PDA-coated polystyrene surfaces (10 mg/mL), with or without the FAK inhibitor PF573228 (10 µM) or JNK inhibitor SP600125 (30 µM). Images were acquired at 0, 6, 9, 12, and 24 h (original magnification ×40). The wound width was measured at three positions per field at different times. Data are presented as mean ± SD values from three independent experiments. Scale bar = 650 µm.

To assess the involvement of FAK-dependent pathways in cell migration, wound-healing assays were performed following PDA exposure (10 mg/mL). After 24 h, PDA-coated PC3 and DU145 cells exhibited nearly complete wound closure, reaching 98.6% and 99.1% closure, respectively (Figure 8). The inhibition of FAK or JNK effectively suppressed PDA-enhanced migration across all cell lines, indicating that these pathways are preferentially engaged by PDA-induced adhesion signaling (Figure 8).

**Figure 9:**
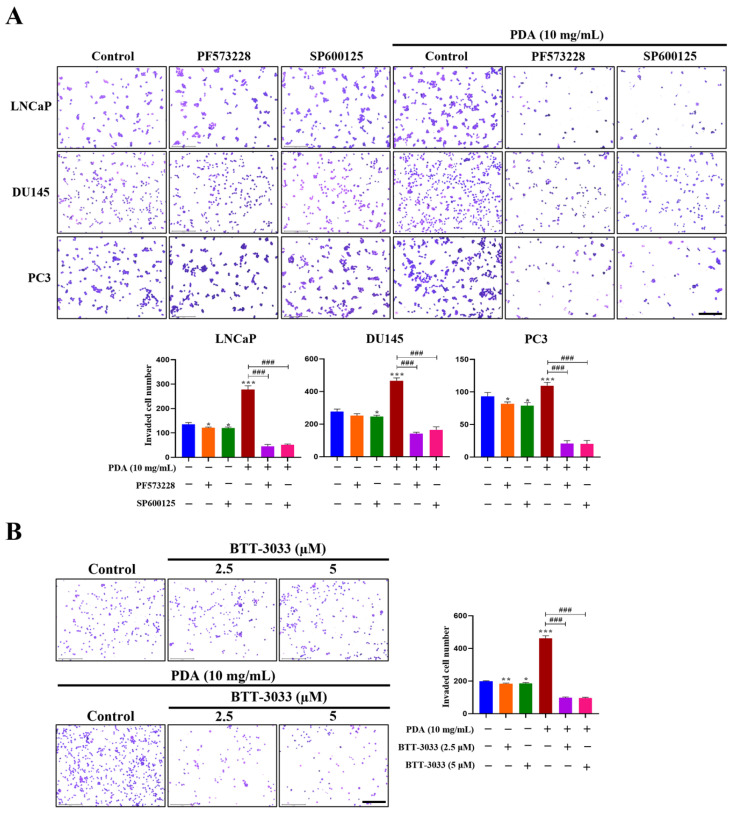
Effects of FAK, JNK, and integrin α_2_β_1_ inhibition on PDA-induced invasion of human PC cells. (**A**) Representative images from hydrogel-based invasion assays of LNCaP, DU145, and PC3 cells cultured on polystyrene surfaces coated with 10 mg/mL PDA, in the presence or absence of the FAK inhibitor PF573228 (10 µM) or the JNK inhibitor SP600125 (30 µM). Images were acquired at 24 h (original magnification ×100), and quantification of cell invasion on PDA-coated polystyrene surfaces assessed by crystal violet staining. (**B**) Representative images of PC3 cells following invasion in the presence or absence of the integrin α_2_β_1_ inhibitor BTT-3033 (2.5 and 5 µM) (original magnification ×100), along with quantification of PC3 cell invasion on PDA-coated surfaces assessed by crystal violet staining. Cells were pretreated with BTT-3033 prior to invasion. Data are presented as mean ± SD from three independent experiments. * *p* < 0.05, ** *p* < 0.01, and *** *p* < 0.001 versus uncoated control; ^###^ *p* < 0.001 versus PDA-treated group. Scale bar = 275 µm.

Hydrogel invasion assays further demonstrated that 10 mg/mL PDA-coated cells displayed a significantly greater invasive capacity than uncoated controls at 20 h (Figure 9). The number of invaded cells under PDA coating conditions relative to control was approximately 3.0-fold higher for LNCaP cells (Control 135.3 ± 7.02; PDA: 278.3 ± 15.04, *p* < 0.001), and 5.0-fold higher for DU145 cells (Control 277.0 ± 15.4; PDA: 466.3 ± 17.24, *p* < 0.001), and 1.2-fold higher for PC3 cells (Control 93.3 ± 5.86; PDA: 109.7 ± 5.03, *p* < 0.05), (Figure 9). This overall increase was most pronounced in LNCaP and DU145 cells and was eliminated by FAK, JNK, or integrin α_2_β_1_ inhibition across all cell lines (Figure 9). Notably, treatment with PF573228, SP600125, and BTT-3033 exerted minimal effects on basal invasion in cells cultured on uncoated control surfaces (Figure 9B). These results indicate that integrin–FAK–JNK signaling is selectively amplified in the context of enhanced cell invasion under PDA-coated conditions, rather than reflecting nonspecific inhibitory effects of the compounds.

Collectively, these findings demonstrated that PDA promotes the adhesion, migration, and invasion of human PC cells via integrin-dependent mechanisms involving its downstream FAK and JNK signaling.

### 2.8. FAK Activation Mediates PDA-Induced Proliferation of Human PC Cells

**Figure 10:**
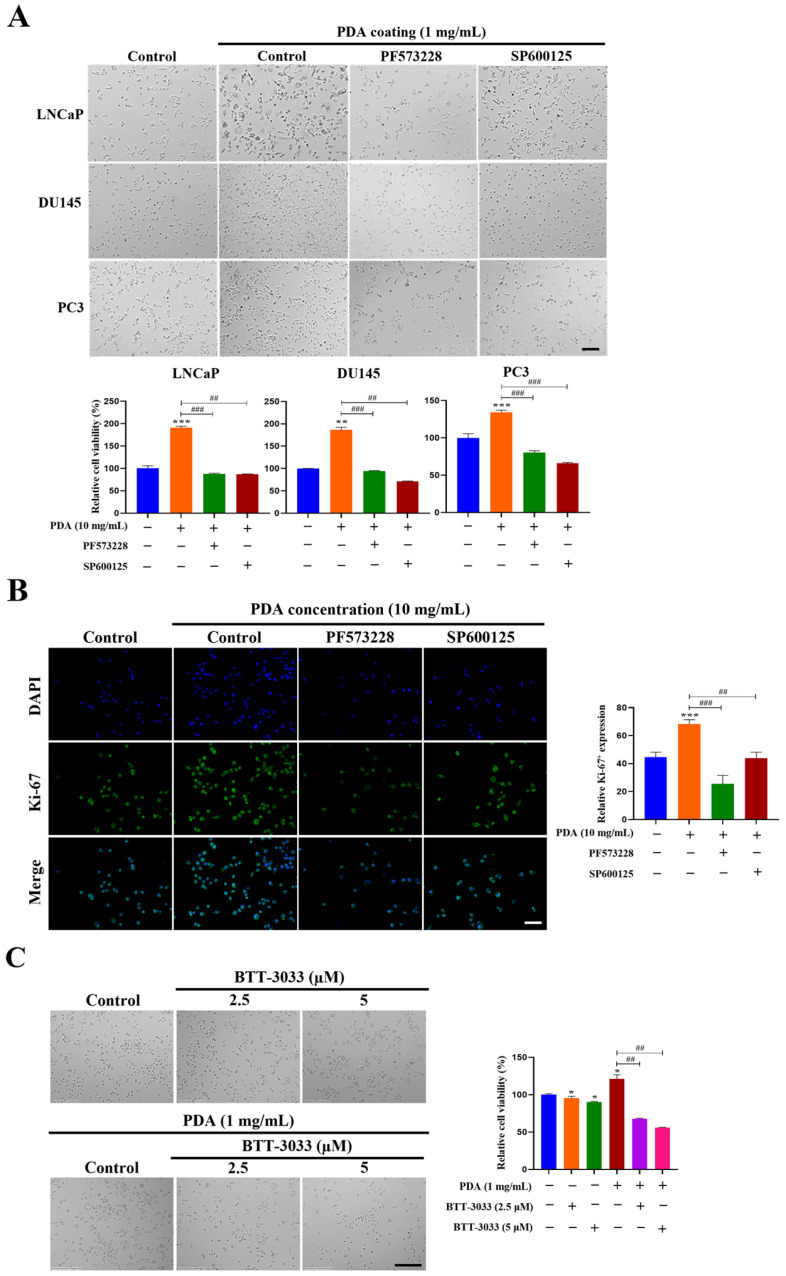

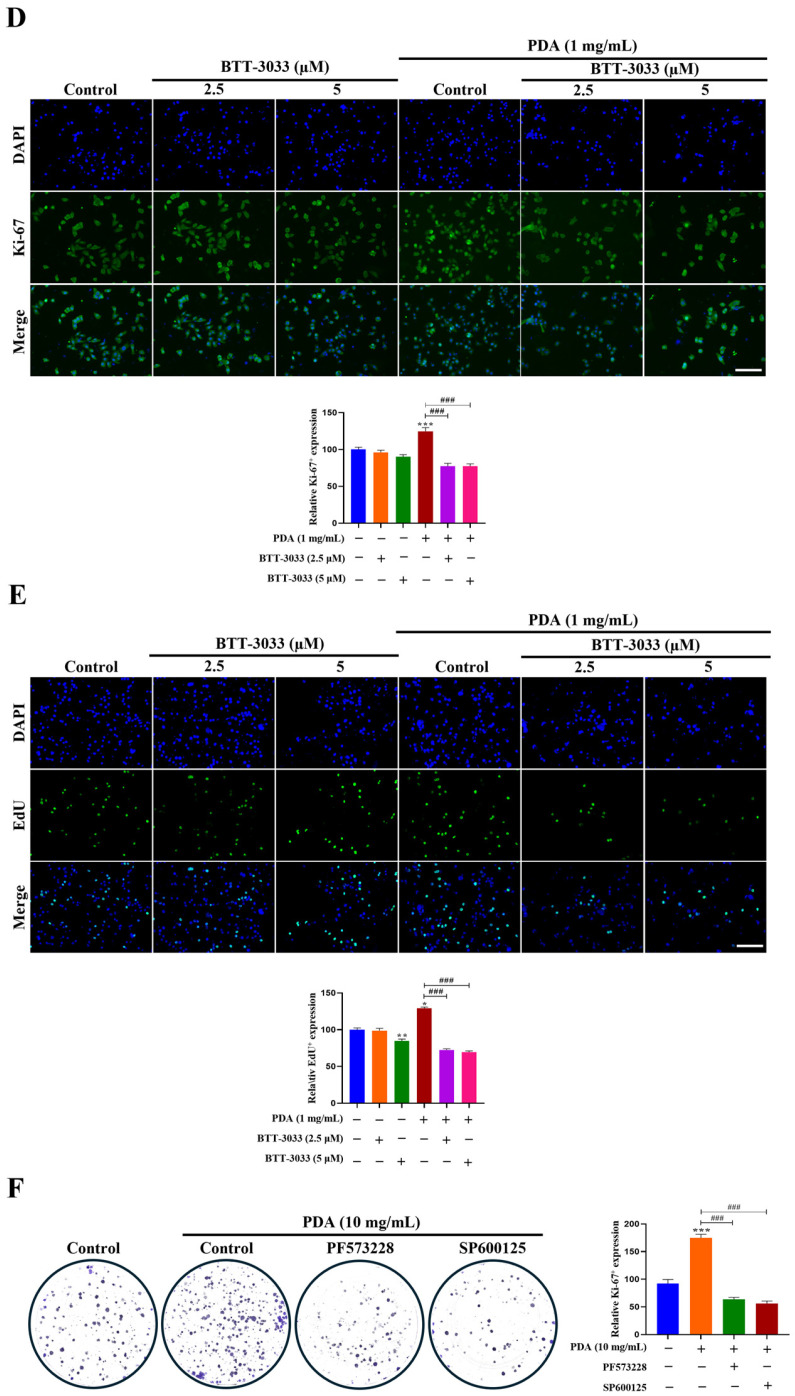
Effects of FAK, JNK, and integrin α_2_β_1_ inhibition on PDA-induced proliferation of human PC cells. (**A**) WST-1 assay results and representative phase-contrast images of LNCaP, DU145, and PC3 cells cultured on polystyrene surfaces coated with PDA (1 mg/mL), in the presence or absence of the FAK inhibitor PF573228 (10 µM) or the JNK inhibitor SP600125 (30 µM) (original magnification ×100; scale bar = 275 µm). (**B**) Representative confocal images of Ki-67 immunofluorescence staining in LNCaP, DU145, and PC3 cells under the same treatment conditions (original magnification ×100; scale bar = 275 µm). (**C**) WST-1 assay results and representative phase-contrast images of PC3 cells cultured on PDA-coated polystyrene surfaces (1 mg/mL), with or without the integrin α_2_β_1_ inhibitor BTT-3033 (2.5 and 5 µM) (original magnification ×100; scale bar = 275 µm). (**D**,**E**) Representative confocal images of Ki-67 (**D**) and EdU (**E**) staining in PC3 cells under the same treatment conditions (original magnification ×100; scale bar = 275 µm). (**F**) Colony-formation assay results for PC3 cells cultured on PDA-coated surfaces for 14 days in the presence or absence of PF573228 or SP600125 (original magnification ×40; scale bar = 650 µm). Data are presented as mean ± SD from three independent experiments. * *p* < 0.05, ** *p* < 0.01, and *** *p* < 0.001 vs. uncoated control; ^##^ *p* < 0.01, ^###^ *p* < 0.001 vs. PDA-treated group.

The LNCaP, DU145, and PC3 cells exhibited significantly increased proliferation after 24 h of incubation on 1 mg/mL PDA-coated surfaces, with 1.3-, 1.9-, and 1.8-fold increases, respectively, compared to the uncoated controls (*p* < 0.001–0.01; Figure 10A). Consistent with these findings, phase-contrast images showed higher cell densities on the PDA-coated surfaces (Figure 10A).

To determine whether the PDA-induced proliferation was mediated by integrin-dependent signaling, cells were cultured on 10 mg/mL PDA-coated surfaces in the presence of pathway inhibitors. Treatment with the FAK inhibitor PF573228 (10 µM) or JNK inhibitor SP600125 (30 µM) suppressed the PDA-enhanced proliferation in the LNCaP, DU145, and PC3 cells (Figure 10B). Confocal imaging further demonstrated an increase in Ki-67 expression following PDA exposure, which was reduced by the FAK or JNK inhibitor (Figure 10B). Notably, treatment with BTT-3033 exerted minimal effects on basal proliferation in cells cultured on uncoated control surfaces, whereas the inhibitory effects of BTT-3033 (2.5 and 5 µM) were markedly more pronounced under PDA-coated conditions (Figure 10C–E). These results indicate that integrin–FAK–JNK signaling is selectively amplified in the context of enhanced cell proliferation under PDA-coated conditions, rather than reflecting nonspecific inhibitory effects of the compounds.

Clonogenic assays revealed that the PDA coating promoted a long-term growth capacity. PC3 cells formed significantly more colonies on PDA-coated surfaces than on uncoated controls after 10 days (174.7 ± 6.5 vs. 92.0 ± 7.5 colonies; *p* < 0.001), indicating enhanced clonogenic potential (Figure 10F).

Collectively, these results demonstrated that PDA stimulates human PC cell proliferation and clonogenicity through integrin-dependent mechanisms involving its downstream FAK and JNK signaling.

### 2.9. PDA Coating Increases Stemness- and Malignancy-Associated Gene Expression via Activation of the FAK–JNK Signaling Pathway

**Figure 11:**
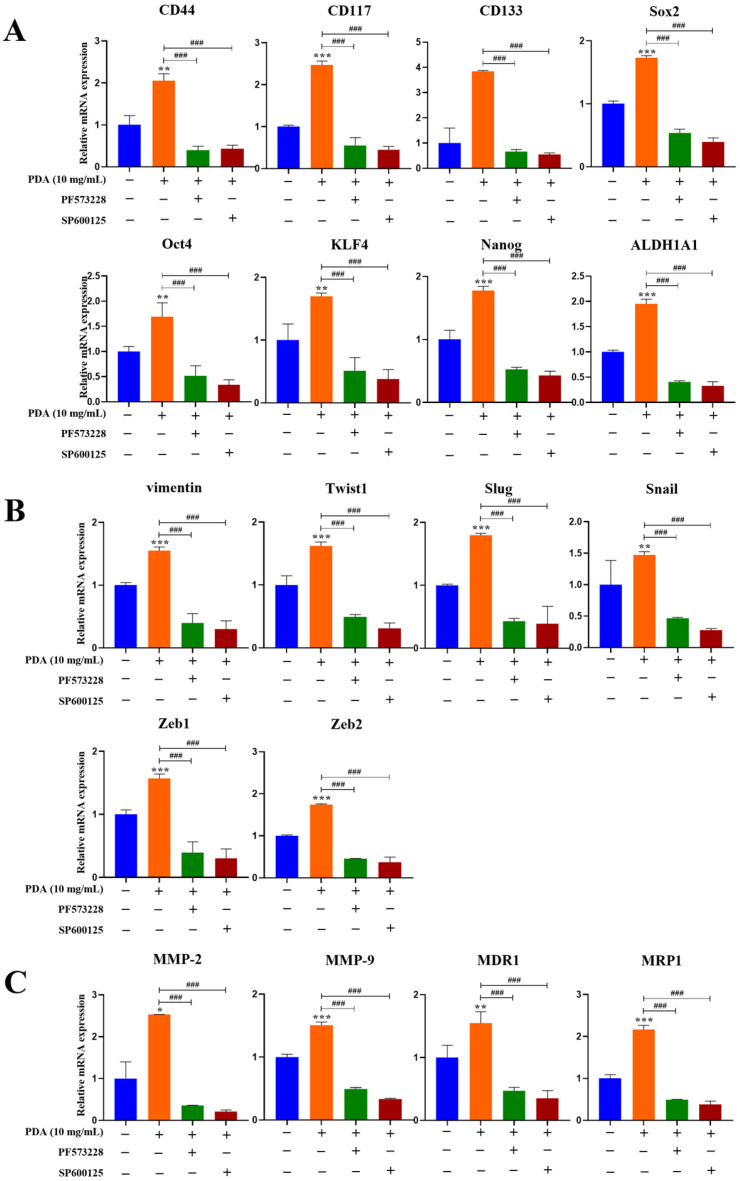

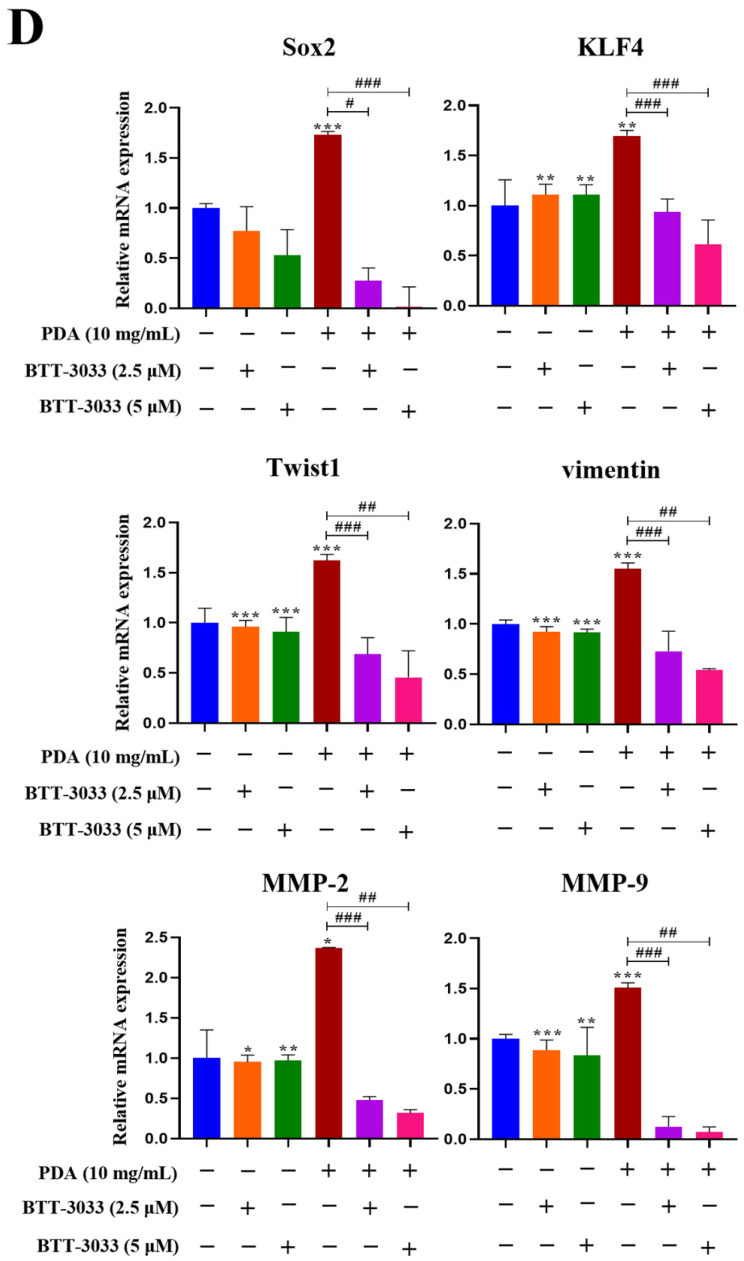
Effects of PDA coating and integrin–FAK–JNK pathway inhibition on stemness-, EMT-, and drug-resistance-associated gene expression in human PC cells. (**A**–**C**) qRT-PCR analysis of mRNA expression levels of stemness-related genes (*CD44*, *CD117*, *CD133*, *Sox2*, *Oct4*, *KLF4*, *Nanog*, and *ALDH1A1*) (**A**), EMT-associated transcription factors (*vimentin*, *Twist1*, *Slug*, *Snail*, *Zeb1*, and *Zeb2*) (**B**), matrix metalloproteinases (*MMP-2*, *MMP-9*) and multidrug resistance-associated genes (*MDR1*, *MRP1*) (**C**) in PC3 cells cultured on PDA-coated polystyrene surfaces (10 mg/mL) for 24 h in the presence or absence of the FAK inhibitor PF573228 (10 µM) or the JNK inhibitor SP600125 (30 µM). (**D**) qRT-PCR analysis of mRNA expression levels of stemness-related genes (*Sox2* and *KLF4*), EMT-associated transcription factors (*Twist1* and *vimentin*), and matrix metalloproteinases (*MMP-2* and *MMP-9*) in PC3 cells cultured on PDA-coated polystyrene surfaces (10 mg/mL) for 24 h, with or without the integrin α_2_β_1_ inhibitor BTT-3033 (2.5 and 5 µM). *GAPDH* was used as an internal control for normalization. Data are presented as mean ± SD from three independent experiments. Statistical significance was determined relative to uncoated control cells (* *p* < 0.05, ** *p* < 0.01, and *** *p* < 0.001 vs. uncoated control; and ^#^ *p* < 0.05, ^##^ *p* < 0.01, and ^###^ *p* < 0.001 vs. PDA-treated cells without inhibitor).

To determine whether FAK–JNK signaling mediates the PDA-induced upregulation of malignancy-associated genes, a qRT-PCR analysis was performed in PC3 cells following adhesion to PDA-coated surfaces (10 mg/mL) for 24 h (Figure 11). The PDA coating significantly increased the expression of EMT-associated transcription factors (*Snail*, *Slug*, *Twist1*, *Zeb1*, *Zeb2*, and *vimentin*), matrix metalloproteinases (*MMP-2* and *MMP-9*), stemness-related markers (*CD44*, *CD133*, *SOX2*, *KLF4*, *ALDH1A1*, and *NANOG*), and multidrug resistance-associated genes (*MDR1* and *MRP1*) compared to the uncoated controls (Figure 11).

Pretreatment with the FAK inhibitor PF573228 (10 µM) or JNK inhibitor SP600125 (30 µM) suppressed the PDA-induced upregulation of these genes, indicating that their expression is dependent on FAK–JNK signaling activation (Figure 11A–C).

Notably, treatment with BTT-3033 exerted minimal effects on basal mRNA expression levels of stemness-related genes (*Sox2* and *KLF4*), EMT-associated transcription factors (*Twist1* and *vimentin*), and matrix metalloproteinases (*MMP-2* and *MMP-9*) in PC3 cells cultured on PDA-coated polystyrene surfaces (10 mg/mL) for 24 h, whereas the inhibitory effects of BTT-3033 (2.5 and 5 µM) on the mRNA expression levels of these key genes associated with malignant phenotypes markedly more pronounced under PDA-coated conditions (Figure 11D). These results indicate that integrin–FAK–JNK signaling is preferentially engaged by PDA-induced malignant gene-expression programs, rather than reflecting nonspecific inhibitory effects of the compounds.

Collectively, these findings demonstrated that PDA stimulates the gene expression of cancer stemness- and malignancy-related molecules in human PC cells through an FAK-dependent mechanism involving downstream JNK signaling.

### 2.10. PDA Coating Increases CSC Markers via FAK–JNK Signaling in Human PC Cells

**Figure 12:**
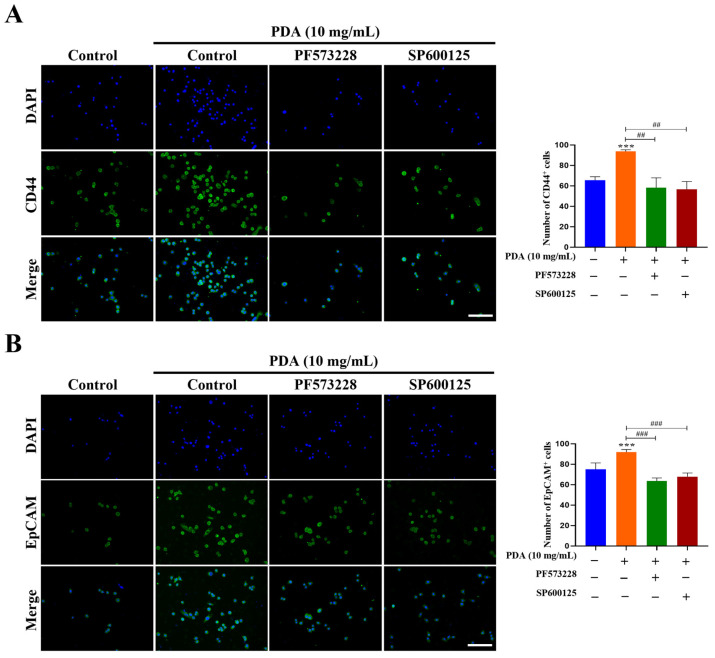
Effects of PDA coating and FAK–JNK inhibition on cancer stem cell marker expression in human PC cells. Confocal images and quantitative analysis of CD44 (**A**) and EpCAM (**B**) expressions in PC3 cells cultured on PDA-coated polystyrene surfaces (10 mg/mL) for 24 h, in the presence or absence of the FAK inhibitor PF573228 (10 µM) or the JNK inhibitor SP600125 (30 µM) (original magnification ×100). PDA-coated surfaces increased the CD44 and EpCAM expression compared with uncoated control surfaces, whereas inhibition of FAK or JNK markedly reduced these effects. Data are presented as mean ± SD from three independent experiments. Statistical significance was determined relative to uncoated control cells. *** *p* < 0.001 vs. uncoated control; ^##^ *p* < 0.01, ^###^ *p* < 0.001 vs. PDA-treated cells without inhibitor. Scale bar = 275 µm.

Consistent with the qRT-PCR findings shown in Figure 11, confocal imaging showed increased expressions of the CSC-associated surface markers CD44 and EpCAM in PC3 cells after 24 h on PDA-coated surfaces (10 mg/mL) compared to uncoated controls (1.4- and 1.2-fold increases, respectively; *p* < 0.001–0.05) (Figure 12). These effects were suppressed by pretreatment with the FAK inhibitor PF573228 (10 µM) or JNK inhibitor SP600125 (30 µM) (Figure 12).

Collectively, these findings indicate that PDA enhances CSC-like phenotype in human PC cells via an FAK-dependent mechanism involving downstream JNK signaling.

Notably, these PDA-induced phenotypic changes were observed across PC cell lines representing distinct androgen receptor statuses, indicating that PDA-driven integrin–FAK–JNK signaling operates independently of androgen receptor expression.

### 2.11. PDA Coating Increases Chemoresistance in Human PC Cells

Multidrug resistance remains a major barrier to effective PC therapy. Previous studies have shown that surface preconditioning with ECM components can alter the drug sensitivity of cancer cells [70]. Given our findings that PDA promotes adhesion, migration, and proliferation, which are hallmarks of aggressive tumor behavior, we hypothesized that PDA-coated surfaces would enhance the chemoresistance of human PC cells.

**Figure 13:**
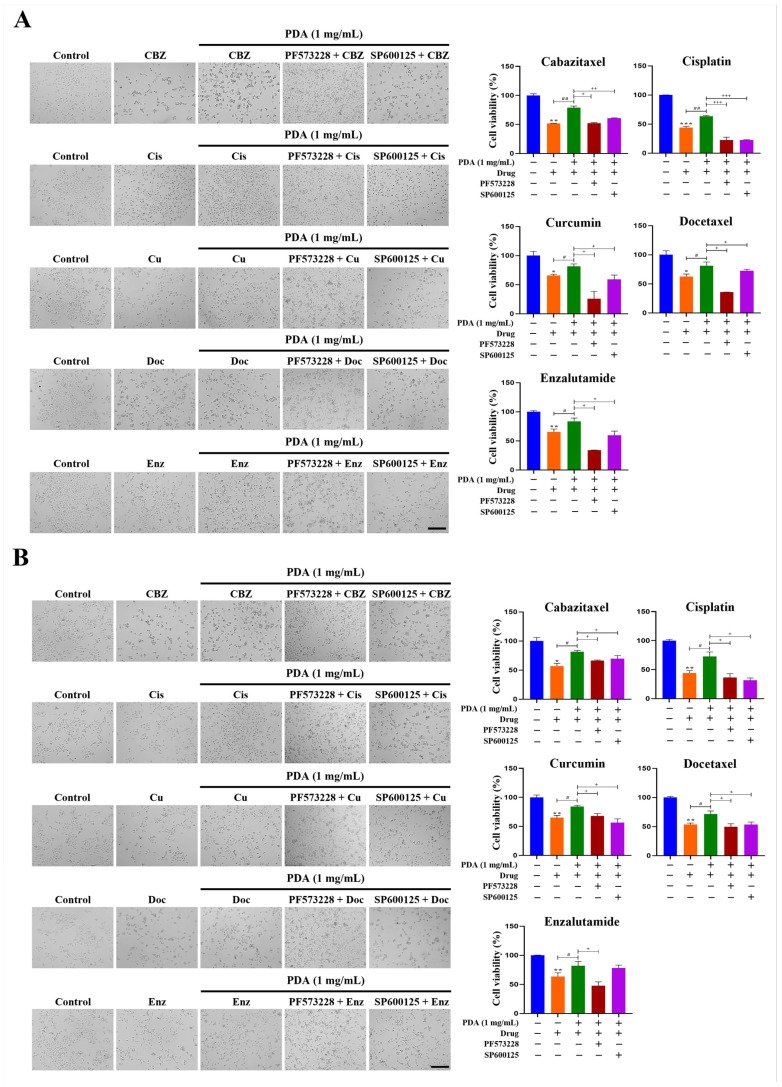

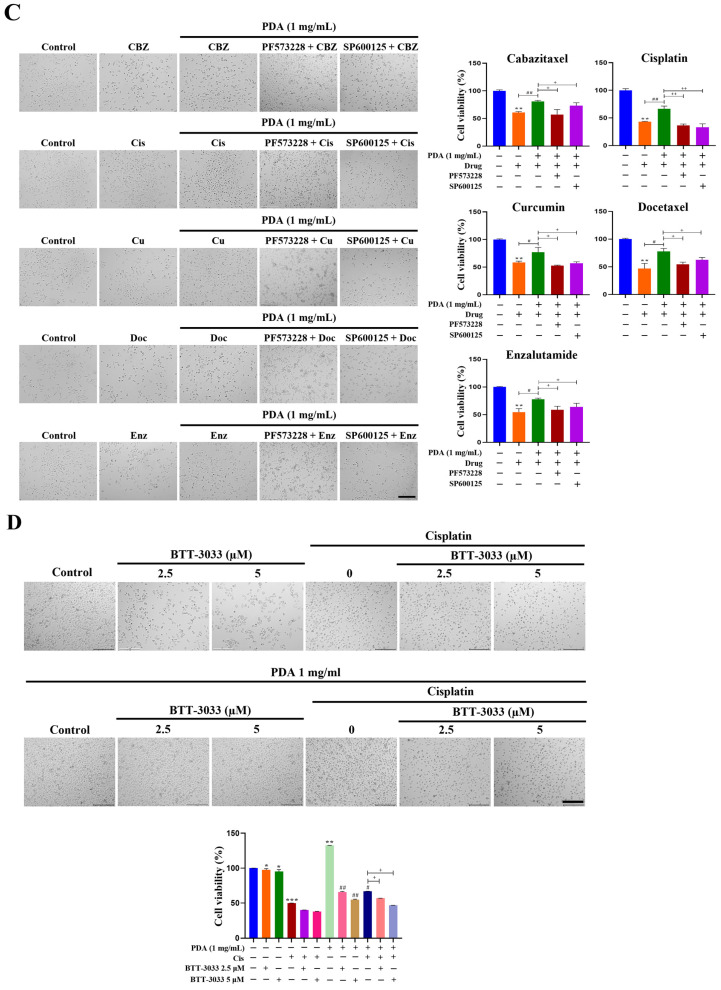
Effects of PDA coating and integrin–FAK–JNK pathway inhibition on chemosensitivity of human PC cells, but this effect is reversed by FAK, JNK, or integrin α_2_β_1_ inhibition. (**A**–**C**) WST-1 cytotoxicity assays showing viability of LNCaP (**A**), DU145 (**B**), and PC3 (**C**) cells cultured on PDA-coated polystyrene surfaces (1 mg/mL) following treatment with cabazitaxel (10 nM), cisplatin (100 µM), curcumin (50 µM), docetaxel (200 nM), or enzalutamide (25 µM) for 24 h, in the presence or absence of the FAK inhibitor PF573228 (10 µM) or the JNK inhibitor SP600125 (30 µM) (original magnification ×100). (**D**) WST-1 cytotoxicity assays showing viability of PC3 cells cultured on PDA-coated polystyrene surfaces (10 mg/mL) following treatment with cisplatin (100 µM) for 24 h, with or without the integrin α_2_β_1_ inhibitor BTT-3033 (2.5 and 5 µM). Representative phase-contrast images corresponding to each condition are shown (original magnification ×100). Data are presented as mean ± SD from three independent experiments performed in triplicate. Statistical significance was determined relative to uncoated control cells (* *p* < 0.05, ** *p* < 0.01, and *** *p* < 0.001 vs. uncoated control; ^#^ *p* < 0.05, and ^##^ *p* < 0.01 vs. PDA-coated control; and ^+^ *p* < 0.05, ^++^ *p* < 0.01, and ^+++^ *p* < 0.001 vs. PDA-coated + drug-treated group; scale bar = 275 µm).

To test this, cytotoxicity assays were performed on LNCaP, DU145, and PC3 cells cultured on polystyrene surfaces coated with 1 mg/mL PDA for 24 h. WST-1 analysis revealed significantly reduced sensitivities to multiple antitumor agents compared with uncoated controls (Figure 13).

Increases in chemoresistance were observed in LNCaP cells, ranging from 1.3- to 1.8-fold compared to uncoated controls (Figure 13A). For example, treatment with cisplatin (100 µM) resulted in a 1.8-fold increase (*p* < 0.05), and cabazitaxel (10 nM) resulted in a 1.6-fold increase (*p* < 0.05). Similar increases in chemoresistance were observed in DU145 cells, chemoresistance was similarly enhanced (1.3- to 1.8-fold; Figure 13B). Treatment with cisplatin (100 µM) and docetaxel (200 nM) both showed 1.8-fold increases (*p* < 0.01 and *p* < 0.05, respectively; Figure 13B).

In PC3 cells, the PDA coating increased the cell viability following treatment with cabazitaxel (10 nM), cisplatin (100 µM), curcumin (50 µM), docetaxel (200 nM), and enzalutamide (25 µM) by 1.4- (*p* < 0.01), 1.5- (*p* < 0.01), 1.3- (*p* < 0.05), 1.3- (*p* < 0.05), and 1.3-fold (*p* < 0.05), respectively (Figure 13C).

Cells grown on the uncoated surfaces served as a reference for 100% viability. The cytotoxicity profiles corresponded with morphological observations using phase-contrast microscopy, which showed visibly preserved cell densities on the PDA-coated surfaces following drug exposure (Figure 13A–C).

Importantly, treatment with the FAK inhibitor PF573228 (10 µM) and JNK inhibitor SP600125 (30 μM) effectively abolished the PDA-induced chemoresistance in all three PC cell lines (Figure 13A–C), indicating pathway dependence.

Notably, treatment with the integrin α_2_β_1_ inhibitor BTT-3033 exerted minimal effects on basal chemoresistance in cells cultured on uncoated control surfaces, whereas its inhibitory effects (2.5 and 5 µM) were markedly more pronounced under PDA-coated conditions (Figure 10C–E). These findings indicate that integrin-dependent signaling is preferentially engaged under PDA-induced conditions, rather than reflecting nonspecific inhibitory effects of the compound.

In addition, the reduced drug sensitivity observed on PDA-coated surfaces is unlikely to result from increased proliferation alone, as all anticancer agents tested (cabazitaxel, docetaxel, cisplatin, curcumin, and enzalutamide) are well-established cytotoxic or cytostatic drugs that suppress PC cell viability primarily through induction of cell death or inhibition of survival signaling. Consistent with a bona fide chemoresistance mechanism, PDA exposure significantly upregulated canonical multidrug resistance genes, including *MDR1* and *MRP1*.

Importantly, pharmacological inhibition of integrin α_2_β_1_, FAK, or JNK fully restored chemosensitivity under PDA-coated conditions. Collectively, these results demonstrate that PDA coating enhances resistance to multiple antitumor agents in human PC cells through activation of integrin-dependent FAK–JNK signaling.

## 3. Discussion

PC remains one of the most frequently diagnosed malignancies in men, and its progression to therapy-resistant and metastatic diseases continues to drive clinical mortality [71]. Although the molecular drivers of PC have been increasingly characterized, the lack of physiologically relevant in vitro systems limits mechanistic investigations and preclinical evaluations [72,73]. Conventional 2D monolayer cultures fail to recapitulate key aspects of the in vivo tumor microenvironment, including three-dimensional (3D) architecture, cell–cell and cell–extracellular matrix (ECM) interactions, biochemical and biomechanical gradients, mechanotransduction, spatial organization, and adhesion-dependent signaling [74,75,76,77,78,79,80,81]. Consequently, malignant phenotypes such as EMT, stemness, invasion, and drug resistance are often underestimated or lost in standard culture systems [78,82,83,84,85,86,87,88,89]. In contrast, 3D tumor models such as spheroids and organoids more closely resemble in vivo tumor physiology and frequently exhibit malignant phenotypes, including EMT, cancer stemness, metastatic capacity, and therapeutic resistance, even though stromal co-culture is often required to fully reproduce in vivo-like EMT and cancer stem cell (CSC) phenotypes driven by tumor–stroma crosstalk [79,90,91,92].

Advances in 3D culture technologies have enabled more physiologically relevant models for investigating tumor progression and drug resistance [93]. These models are increasingly used to study tumor growth, invasion, metastasis, EMT, CSC biology, and therapeutic responses across multiple cancer types [94,95,96,97,98,99,100,101,102]. Among them, multicellular tumor spheroids are widely applied because they recapitulate essential features of real tumors such as cell–cell and cell–ECM interactions, spatial gradients of nutrients and oxygen, and restricted drug penetration [90,91,103,104,105,106,107,108]. However, despite their high physiological relevance, 3D culture systems often require specialized platforms and present technical challenges, including complex fabrication processes and extended culture periods, leading to increased cost and experimental burden [78,91,102,103,108,109,110,111,112,113,114]. Consistent with these observations, we have previously demonstrated that biomimetic 3D hydrogel-based tumor models reproduce key in vivo tumor characteristics across multiple cancer types, including enhanced EMT, migration, invasion, CSC enrichment, and drug resistance [82,85,88,89,115].

Against this background, a key finding of the present study is that a simple PDA-based surface modification is sufficient to induce multiple malignant phenotypes in human PC cell lines, including enhanced adhesion, migration, invasion, proliferation, clonogenicity, EMT activation, CSC marker expression, and chemoresistance. This rapid acquisition of aggressive traits on PDA-coated surfaces suggests that biochemical and adhesive cues alone—without a 3D architecture or exogenously applied ECM gels—can profoundly modulate tumor cell behavior. Importantly, and in direct response to concerns regarding physiological relevance, PDA-based culture systems employed here are not intended to mimic patient tumors or the full in vivo tumor microenvironment, but rather serve as a reductionist and controllable in vitro platform to interrogate how surface-derived adhesive cues influence PC cell plasticity and associated signaling programs.

Although PDA is not a native ECM component, its catechol- and amine-rich chemistry enables robust and non-selective adsorption of serum- or cell-derived ECM proteins—including collagens, fibronectin, and vitronectin—thereby enhancing integrin engagement in a reproducible manner [3,116,117,118,119]. Accordingly, unlike single-ECM coatings such as collagen I or fibronectin, which selectively activate specific integrin subsets, PDA provides a broad adhesive interface that promotes adsorption of diverse ECM proteins and engagement of multiple integrin receptors, enabling investigation of global adhesion signaling rather than ligand-restricted pathways [23,120,121]. Notably, the phenotypes induced by PDA-coated surfaces in the present study—enhanced adhesion, migration, invasion, proliferation, EMT, CSC marker expression, and chemoresistance—are strikingly consistent with those observed in ECM-rich or 3D tumor models [79,90,91,93,94,95,96,97,98,99,100,101,102,103], including our previously reported biomimetic 3D prostate cancer system [88]. These parallels support the interpretation that PDA-based culture can capture key functional consequences of ECM-mediated/3D contexts, despite lacking higher-order tissue architecture.

Mechanistically, we identified the activation of the integrin α_2_β_1_–FAK signaling pathway as central mediators of PDA-dependent responses, with downstream engagement of JNK signaling. Integrin α_2_β_1_ is a key collagen receptor, with high affinity for type I collagen [122]. Integrin α_2_β_1_ mediates firm adhesion to collagen-rich ECM and transduces “outside-in” and “inside-out” signals that regulate cytoskeletal organization, survival, proliferation, migration, and invasion by activating canonical integrin signaling nodes, including FAK [123,124]. Hall et al. [125,126] showed that PC cells derived from skeletal metastases express higher levels of α_2_β_1_, adhere strongly to collagen I, and display enhanced chemotaxis toward collagen. Ziaee & Chung [127] reported induction of *integrin α*_2_ together with a broader pro-metastatic transcriptional program, including MMPs and other invasion-related genes, supporting a role for α_2_β_1_ in promoting colonization and growth within bone in a highly bone-metastatic PC3 subline (PC3-N2). Integrin α_2_β_1_ is also linked to PC stem/progenitor populations. Ojalill et al. [128] demonstrated that α_2_β_1_^high^ PC cells are enriched in docetaxel-resistant populations and display stem-cell-like traits, including enhanced survival, migration, invasion, and pro-invasive gene expression, suggesting that α_2_β_1_^high^ cells represent a therapy-resistant, highly invasive subpopulation with strong metastatic potential.

In addition, we observed a transient expression pattern of integrin α_2_β_1_ under PDA-coated conditions. Similar temporal dynamics of integrin signaling have been observed in other systems, in which phosphorylation of focal adhesion components and downstream signaling activity increases at early adhesion time points and subsequently attenuates as adhesion complexes mature [129]. More broadly, integrin-mediated mechanotransduction is characterized by rapid initiation followed by modulation of gene expression as cell–substrate interactions stabilize and signaling transitions from an induction phase to a maintenance phase [130]. These dynamics indicate regulated adhesion signaling rather than nonspecific cellular adaptation to the substrate.

Following integrin α_2_β_1_ ligation, FAK serves as a key signaling node linking ECM engagement to cytoskeletal remodeling, transcriptional reprogramming, migration, invasion, metastasis, and chemoresistance [123,131,132]. Aberrant FAK activation is associated with EMT, characterized by upregulation of transcription factors such as Snail, Slug, and Twist, loss of epithelial markers, and acquisition of mesenchymal traits, thereby facilitating local invasion and metastatic spread [133,134,135]. In PC, FAK overexpression correlates with invasive behavior and poor prognosis, and pharmacologic FAK inhibition suppresses tumor cell motility and enhances the efficacy of taxane-based chemotherapy, implicating FAK as a mediator of both metastatic progression and therapy resistance [44,136,137,138,139,140]. The JNK pathway is an important downstream effector of integrin and FAK signaling and further contributes to malignant progression [141]. Integrin β_1_–FAK signaling can activate JNK, which in turn promotes AP-1-dependent transcription of invasion- and EMT-associated genes, including *MMP-2*, *MMP-9*, and *vimentin* [69,142]. Furthermore, in PC and other solid tumors, JNK activity has been shown to support EMT, stemness, and vasculogenic mimicry, and JNK inhibition reverses EMT programs or sensitizes tumor cells to cytotoxic treatment [48,143,144,145,146,147,148]. Notably, integrin β_1_–FAK–JNK signaling has been directly linked to resistance to radiotherapy and chemotherapy, where integrin engagement enhances DNA damage repair and pro-survival signaling, thereby conferring adhesion-mediated therapy resistance [142,149]. Taken together, current evidence supports a model in which integrin α_2_β_1_ engagement of collagen-rich matrices activates FAK and JNK signaling cascades to promote invasion, EMT, and resistance to therapy in PC, positioning the α_2_β_1_–FAK–JNK axis as a promising target for therapeutic intervention.

In this study, pharmacological inhibition of the integrin α_2_β_1_–FAK–JNK signaling axis effectively abolished PDA-induced adhesion, migration, invasion, proliferation, stemness- and EMT-associated gene expression, and chemoresistance, supporting the conclusion that PDA-enhanced malignant traits are dependent on integrin α_2_β_1_–FAK–JNK signaling. These findings are consistent with previous reports implicating this signaling axis in tumor invasion, EMT, and resistance to therapy, and extend this understanding by demonstrating that a biomaterial-derived surface cue can robustly engage integrin-driven signaling in PC cells [123,150,151,152,153,154].

A potential confounding factor in interpreting drug-response assays is whether reduced drug efficacy reflects true chemoresistance or merely altered proliferation rates. In the present study, several lines of evidence support the conclusion that PDA induces a genuine chemoresistant phenotype rather than a proliferation-driven artifact. First, the anticancer agents used are cytotoxic or cytostatic and do not promote proliferation. Second, PDA-induced drug resistance was accompanied by upregulation of established multidrug resistance genes (*MDR1* and *MRP1*), which are mechanistically linked to drug efflux and survival signaling. Third, inhibition of integrin α_2_β_1_, FAK, or JNK selectively reversed PDA-associated resistance while exerting minimal effects on basal proliferation under uncoated conditions. Together, these findings indicate that PDA promotes chemoresistance through integrin-dependent signaling pathways rather than through nonspecific increases in cell growth.

An additional limitation of the present study is that androgen receptor (AR) signaling was not directly interrogated. Although AR signaling is a central driver of PC biology, particularly in androgen-dependent disease, the PDA-induced phenotypic changes described here were observed consistently across both AR-positive (LNCaP) and AR-negative (DU145 and PC3) PC cell lines. This suggests that PDA-mediated enhancement of adhesion, EMT, stemness, and chemoresistance occurs largely independently of AR status and is driven primarily by integrin-dependent signaling mechanisms. Nevertheless, future studies will be required to determine whether PDA exposure modulates AR expression, localization, or transcriptional activity in AR-positive models and how integrin–FAK–JNK signaling may intersect with AR-driven pathways in more physiologically complex systems.

Importantly, inhibition of integrin α_2_β_1_, FAK, or JNK exerted minimal effects on basal cellular behaviors under uncoated conditions, while robustly suppressing PDA-enhanced phenotypes. Together, these data support a model in which PDA selectively amplifies integrin-initiated signaling that propagates through the FAK–JNK axis, rather than reflecting nonspecific inhibitory effects of the compounds. Although basal-control experiments for FAK and JNK inhibition were performed for adhesion and invasion assays, we acknowledge that comparable basal controls were not conducted across all functional endpoints due to experimental constraints; nevertheless, the consistent integrin-level specificity demonstrated by α_2_β_1_ inhibition supports a PDA-dependent amplification of this signaling axis.

From an application standpoint, PDA coating offers a practical approach to generate a robust adhesion-driven phenotype shift in standard culture plates, which may facilitate mechanistic studies of EMT/stemness programs and preliminary assessment of drug responses under defined adhesive conditions. However, PDA-coated surfaces should be viewed as complementary to, rather than substitutes for, physiologically complex 3D and patient-derived models, and validation in such systems will be required to establish translational relevance and to capture stromal, immune, and vascular contributions to tumor progression. In addition, the potential influence of PDA oxidation state, coating thickness, and substrate composition on cancer behavior warrants further evaluation.

In summary, our results revealed that a PDA coating is a potent regulator of PC cell behavior through the activation of integrin-dependent FAK–JNK signaling. Beyond providing new insights into biomaterial–tumor cell interactions, this study introduced a simple and scalable in vitro platform for dissecting adhesion-mediated signaling and phenotypic plasticity in PC cell lines, while recognizing that further validation in 3D and patient-derived models will be necessary to bridge mechanistic findings with physiological relevance.

## 4. Materials and Methods

### 4.1. Cell Culture and Reagents

Human PC cell lines (LNCaP, DU-145, and PC3) were purchased from the American Type Culture Collection (ATCC, Manassas, VA, USA). All of the cell lines were cultured and maintained in RPMI 1640 (Hyclone, Chicago, IL, USA) and DMEM (Hyclone, Chicago, IL, USA), supplemented with 10% fetal bovine serum (FBS, Welgene, Daegu, Republic of Korea) and 1% penicillin-streptomycin (Gibco/Thermo Fisher Scientific, Carlsbad, CA, USA) in a 5% CO_2_ humidified atmosphere at 37 °C. The medium was changed every third day.

### 4.2. Synthesis of PDA Solution and Surface Coating

PDA solutions were prepared by dissolving dopamine hydrochloride (Sigma-Aldrich, St. Louis, MO, USA) in 10 mM Tris–HCl buffer (pH 8.0) at the desired concentrations. The solution was vortexed until complete dissolution was achieved and then allowed to undergo spontaneous oxidative polymerization, as indicated by a gradual color change from colorless to dark brown.

The PDA solution was immediately dispensed into polystyrene cell culture plates to uniformly cover the surface. The plates were wrapped in aluminum foil to minimize light exposure and incubated at room temperature on an orbital shaker for 2 h to allow PDA deposition. Following incubation, the PDA-coated plates were thoroughly washed with phosphate-buffered saline (PBS) to remove unbound polymer and residual reagents. The coated surfaces were then dried under ultraviolet (UV) light overnight prior to subsequent experimental use.

PDA concentrations used for individual assays were determined based on preliminary optimization experiments. Specifically, adhesion assays were first performed across a wide concentration range (0–30 mg/mL) to identify a concentration that produced robust, near-saturating enhancement of cell attachment. Based on these results, 10 mg/mL PDA was selected for subsequent adhesion-dependent assays, whereas a lower concentration (1 mg/mL) was used for proliferation assays to avoid saturation effects and allow accurate quantification of changes in cell growth.

### 4.3. Cell Adhesion Assay

PC cells were seeded at a density of 1 × 10^4^ cells per well in 96-well polystyrene cell culture plates (SPL Life Sciences, Pocheon, Republic of Korea) on uncoated (control) or PDA-coated surfaces and allowed to adhere for 5, 15, 30, 45, 60, 90, and 120 min. Non-adherent cells were gently removed by washing with PBS, while the adhered cells under each condition were fixed with 4% paraformaldehyde for 15 min at room temperature (RT) and washed with PBS. The cells were then stained with 0.1% crystal violet (Sigma-Aldrich) for 15 min. After the second PBS wash, the stained cells were counted to determine the number of attached cells. Each experiment was repeated thrice. Images were captured using an EVOS M7000 imaging system (Thermo Fisher Scientific) at each incubation time from three separate wells, and the stained cells were counted using Celleste Image Analysis Software v5.0 (Thermo Fisher Scientific).

### 4.4. Wound Healing Assay

PC cells were seeded overnight at a density of 1 × 10^6^ cells on uncoated or PDA-coated (0, 1, 5, and 10 mg/mL) surfaces in 6-well polystyrene cell culture plates (SPL Life Sciences). After reaching complete confluency, the cells were scraped with a scratcher (SPL Life Sciences), and the medium was changed. After creating wounds in each well, the wells were washed with DPBS to remove cellular debris and avoid the re-establishment of displaced cells. Wound closure was monitored and images were obtained at 0, 3, 5, 7, 9, 12, and 24 h using an EVOS 7000 (Thermo Fisher Scientific) fully automated imaging system. The images were analyzed using the Celleste Image Analysis Software v5.0 (Thermo Fisher Scientific). Each experiment was repeated thrice.

### 4.5. Hydrogel Invasion Assay

The invasive capacity of the PC cells was assessed using a hydrogel-based invasion assay in 96-well plates (SPL Life Sciences). Briefly, 1 × 10^4^ cells/well were harvested, resuspended in RPMI 1640 (Hyclone) or DMEM (Hyclone), and seeded onto the upper surfaces of the MCP-B hydrogel-coated wells. The lower compartment contained medium supplemented with 10% FBS (Welgene) and 1% penicillin–streptomycin (Thermo Fisher Scientific) as chemoattractants. The cells were incubated at 37 °C in a humidified atmosphere containing 5% CO_2_. After 20 h, the hydrogels were carefully removed by sectioning, and the cells that had invaded through and adhered to the bottom of the wells were fixed with 100% methanol at −20 °C. The fixed cells were stained with 0.5% crystal violet solution (Sigma-Aldrich) for 10 min and gently washed with PBS. The number of invading cells was quantified by counting five random microscopic fields per well using a phase-contrast microscope (EVOS M7000, Thermo Fisher Scientific). Each experiment was performed in triplicate to ensure reproducibility.

### 4.6. Cell Proliferation Assay

To evaluate the effect of the PDA coating on cell proliferation, a 96-well plate was coated with PDA at a concentration of 1 mg/mL for 2 h. After washing with PBS and drying overnight under UV light, the cells were plated at a density of 6 × 10^3^ cells per well and maintained in RPMI 1640 medium (Hyclone) containing 5% FBS for 24 h. Cell proliferation was assessed using the WST-1 colorimetric assay according to the protocol provided by Daeil Lab Service (Seoul, Republic of Korea). Briefly, after washing the plate with Dulbecco’s phosphate-buffered saline (DPBS), 10 μL of WST-1 reagent was added to each well. The plates were then left to incubate for 1 h in a humidified chamber at 37 °C with 5% CO_2_. The metabolic activity of the cells was determined by measuring the absorbance of formazan at 450 nm using a microplate reader (Tecan, Männedorf, Switzerland). Cell survival was expressed as a percentage relative to untreated controls. The cell morphology was monitored at specified intervals using an EVOS M7000 imaging system (Thermo Fisher Scientific) for phase-contrast microscopy. This procedure was repeated independently at least thrice.

### 4.7. Cytotoxic Activity Assay

Cells were pre-treated 1 h before seeding on PDA-modified surfaces with inhibitors targeting FAK (PF53766, 10 μM) and JNK (SP600125, 30 μM). Then, they were seeded at a density of 1 × 10^4^ cells/well in 96-well polystyrene cell culture plates (SPL Life Sciences). The cells were maintained in RPMI 1640 medium (HyClone, Logan, UT, USA) containing 5% FBS. The cells were then treated with cabazitaxel (10 nM), cisplatin (100 µM), curcumin (50 µM), docetaxel (200 nM), and enzalutamide (25 µM) for 24 h. Cell proliferation was assessed using the WST-1 colorimetric assay according to the protocol provided by Daeil Lab Service (Seoul, Republic of Korea). The metabolic activity of the cells was determined by measuring the absorbance of formazan at 450 nm using a microplate reader (Tecan). Cell survival was expressed as a percentage relative to untreated controls. The cell morphology was monitored using an EVOS M7000 imaging system (Thermo Fisher Scientific). This procedure was repeated independently at least thrice.

### 4.8. RNA Isolation and cDNA Synthesis

The total RNA of the cells was isolated using TRIzol reagent (Favorgen Biotech Corp., Pingtung, Taiwan). The RNA quality and quantity were determined using a NanoDrop 2000 spectrophotometer (Thermo Scientific, Waltham, MA, USA). First-strand cDNA was synthesized with 1 µg total RNA from each sample using a HiSenScript™ RH (−) RTase cDNA Synthesis Kit (iNtRON Biotechnology, Sungnam, Republic of Korea). The reaction mixture was incubated at 45 °C for 60 min and then heated to 85 °C for 10 min to stop the reaction.

### 4.9. Quantitative Real-Time PCR (qRT-PCR)

The Bio-Rad CFX Connect Real-Time system (Hercules, CA, USA) and DNA-binding dye SYBR Green I with the SsoAdvanced Universal SYBR Green Supermix (Bio-Rad) were used for the qRT-PCR. The primers used for the qRT-PCR are listed in Table 1. All of the samples were amplified in triplicates. The gene expression levels were calculated using the 2^−ΔΔCt^ method and normalized to *GAPDH* expression levels. The expression of the control sample was set to 1, and the relative expressions of the other samples were calculated accordingly.

### 4.10. Western Blot Analysis

To measure the protein expression levels, the cells were harvested and washed twice with ice-cold PBS. The cells were then lysed in RIPA lysis buffer (GenDEPOT, Barker, TX, USA) containing a protease inhibitor cocktail (GenDEPOT) for 30 min on ice with agitation by vortex at 10 min intervals. The resulting homogenates were centrifuged at 14,000 rpm for 10 min at 4 °C, and the supernatants were collected. The protein concentration was determined using the bicinchoninic acid (BCA) protein assay (Sigma-Aldrich). An equal amount of protein per sample was mixed with Laemmli sample buffer (Bio-Rad) and loaded (18 μg/lane) into a 4–12% (*v*/*v*) SDS-polyacrylamide gel electrophoresis (PAGE) gel. The separated proteins were blotted onto a polyvinylidene fluoride (PVDF) membrane (Amersham Biosciences, Piscataway, NJ, USA) using the semi-dry transfer method (Bio-Rad). The membranes were blocked with 3% bovine serum albumin (BSA) in tris-buffered saline containing 0.1% Tween-20 at room temperature for 1 h. The membranes were washed with 1× Tris-Buffered Saline with Tween-20 (TBST) three times and incubated overnight at 4 °C with primary antibodies against Sox2 (Abcam, Cambridge, UK) and vimentin (Santa Cruz Biotechnology, Dallas, TX, USA). Subsequently, they were incubated for 1 h at room temperature with anti-rabbit and anti-mouse secondary antibodies (Cell Signaling Technology, Danvers, MA, USA). Following washing, the proteins were visualized using an enhanced chemiluminescence (ECL) kit (Amersham Biosciences) according to the manufacturer’s instructions. Images were captured using an Amersham Imager 680 (Amersham Biosciences) and quantified using ImageJ software (v1.52a, National Institute of Health, Bethesda, MD, USA).

### 4.11. Flow Cytometry

Flow cytometry was used to assess the PC cell adhesion via integrin/FAK in PDA-coated polystyrene plates. Single-cell suspensions were prepared (1 × 10^5^ cells/tube) and washed with Hanks’ balanced salt solution (HBSS, Gibco/Thermo Fisher Scientific) containing 0.1% BSA and 0.1% sodium azide. Cells were stained with purified α_2_β_1_ monoclonal antibody (1:100; Abcam, Cambridge, UK) for 2 h and then stained with an anti-mouse Alexa Fluor 488-conjugated secondary antibody (1:1000; Invitrogen, Life Technologies, Carlsbad, CA, USA) for 1 h at RT in a dark place. To assess the PDA proliferation, 1 × 10^5^ cells/tube were collected, fixed using Cytofix/Cytopermsolution (BD Biosciences, San Jose, CA, USA), and permeabilized using Perm/Wash solution (BD Biosciences). The cells were washed and stained with purified monoclonal Ki-67 (1:100; Invitrogen) for 2 h, followed by incubation with an anti-mouse Alexa Fluor 488-conjugated secondary antibody (1:1000; Invitrogen) for 1 min at RT. The samples were analyzed using a FACS Canto-II flow cytometer (BD Biosciences), and the data were processed using FlowJo 10.3.0 (Tree Star; Ashland, OR, USA).

### 4.12. Evaluation of Immunofluorescence Using Confocal Microscopy

PC cells were cultured on a PDA-coated plate for 24 h at 37 °C in a 5% CO2 humidified incubator. The cells were then washed twice with PBS, fixed with cold 4% paraformaldehyde (Sigma-Aldrich) in PBS for 20 min, permeabilized with 0.1% Triton X-100 (Sigma-Aldrich) in PBS for 10 min, and blocked with 2% BSA (Sigma-Aldrich) for 1 h at RT. The primary antibodies purified CD44 monoclonal antibody (1:200; BioLegend, San Diego, CA, USA), purified EpCAM monoclonal antibody (1:200; BioLegend), purified Ki-67 monoclonal antibody (1:200; BioLegend), and purified integrin α_2_β_1_ monoclonal antibody (1:200; Abcam) were added and incubated at 4 °C overnight. Goat anti-rat secondary antibodies labeled with FSD-488 (1:1000; BioActs, Incheon, Republic of Korea), goat anti-mouse secondary antibodies labeled with FSD-488 (1:1000; BioActs), and goat anti-rabbit secondary antibodies labeled with FSD-488 (1:1000; BioActs) were then added and incubated for 1 h. The nucleus was stained with DAPI (Vector Laboratories, Newark, CA, USA). Immunofluorescence staining was performed using the EVOS M7000 imaging system (Thermo Fisher Scientific).

### 4.13. Statistical Analysis

All of the quantitative results were expressed as the mean ± standard deviation (SD) values of at least three independent experiments. Data were analyzed using two-way ANOVA with PDA concentration and incubation time as independent variables, followed by Bonferroni-corrected post hoc comparisons to assess differences between PDA-coated and uncoated control surfaces at individual time points. Values of *p* < 0.05 were considered statistically significant.

## 5. Conclusions

In this study, we demonstrated that a PDA coating significantly enhanced the adhesion, migration, invasion, proliferation, and clonogenic potential of human PC cells. PDA exposure also induced EMT, increased the expression of CSC markers, and promoted chemoresistance. These effects were mediated through integrin α_2_β_1_ upregulation and activation of the FAK–JNK signaling pathway, because the inhibition of FAK or JNK effectively abolished the PDA-induced malignant phenotypes. Notably, the PDA coating facilitated rapid cell adhesion compared to uncoated surfaces.

These findings identify PDA as a potent regulator of PC cell behavior, underscoring the importance of biomaterial–cell interactions in experimental PC models. This study establishes a simple and scalable platform that functionally enhances malignant phenotypes in PC cells, providing a foundation for the development of more efficient PC models and for future applications in cancer cell detection and therapeutic testing aimed at advancing early diagnostic and therapeutic strategies.

## Figures and Tables

**Table 1:** qRT-PCR primer names and their sequences.

Gene	Forward (5′-3′)	Reverse (5′-3′)
*ALDH1A1*	TGTGACAAGGAATATGTGAGCC	TGAGCCCTCAGATTTGACCTG
*CD44*	CTGCCGCTTTGCAGGTGTA	CATTGTGGGCAAGGTGCTATT
*CD117*	CACCGAAGGAGGCACTTACACA	TGCCATTCACGAGCCTGTCGTA
*CD133*	CACTACCAAGGACAAGGCGTTC	CAACGCCTCTTTGGTCTCCTTG
*GAPDH*	GGAGAAGGCTGGGGCTCAT	TGATGGCATGGACTGTGGTC
*Integrin α*_2_	GTTGCTCAGTCAAGGCA	GCCAAACTGTTCACTTGAAGGAC
*Integrin β*_1_	GGATTCTCCAGAAGGTGGTTTCG	TGCCACCAAGTTTCCCATCTCC
*KLF4*	CATCTCAAGGCACACCTGCGAA	TCGGTCGCATTTTTGGCACTGG
*MDR1*	GCTGTCAAGGAAGCCAATGCCT	TGCAATGGCGATCCTCTGCTTC
*MMP-2*	TGACGGTAAGGACGGACTC	ATACTTCACACGGACCACTTG
*MMP-9*	CAGAGATGCGTGGAGAGT	TCTTCCGAGTAGTTTTGG
*MRP1*	CCGTGTACTCCAACGCTGACAT	ATGCTGTGCGTGACCAAGATCC
*NANOG*	CTCCAACATCCTGAACCTCAGC	CGTCACACCATTGCTATTCTTCG
*OCT4*	CTTGAATCCCGAATGGAAAGGG	GTGTATATCCCAGGGTGATCCTC
*SLUG*	CGGGAAAAGCAATCTGAAGAGGG	GATGCGGCTATACAACACTGGC
*SNAIL*	ACTGCAACAAGGAATACCTCAG	GCACTGGTACTTCTTGACATCTG
*Sox2*	GCTACAGCATGATGCAGGACCA	TCTGCGAGCTGGTCATGGAGTT
*Twist1*	GTCCGCAGTCTTACGAGGAG	GCTTGAGGGTCTGAATCTTGCT
*Vimentin*	CAAAGCAGGAGTCCACTGAG	TAAGGGCATCCACTTCACAG
*ZEB1*	TTACACCTTTGCATACAGAACCC	TTTACGATTACACCCAGACTGC
*ZEB2*	GCGATGGTCATGCAGTCAG	CAGGTGGCAGGTCATTTTCTT

## Data Availability

The original contributions presented in this study are included in the article/Supplementary Material. Further inquiries can be directed to the corresponding author.

## Supplementary Materials

The following supporting information can be downloaded at: https://www.mdpi.com/article/10.3390/ijms27020655/s1.

## Abbreviations

PDA	Polydopamine
ECM	Extracellular matrix
2D	Two-dimensional
3D	Three-dimensional
CAMs	Cell adhesion molecules
FAK	Focal adhesion kinase
JNK	c-Jun N-terminal kinase
PC	Prostate cancer
EMT	Epithelial–mesenchymal transition
pFAK	Phosphorylated FAK
CSC	Cancer stem cell
Sox2	Sex determining region Y-box 2
Oct4	Octamer-binding transcription factor-4
NANOG	Homeobox protein NANOG transcription factor
KLF4	Krüppel-like factor 4
ALDH1A1	Aldehyde dehydrogenase 1 family, member A1
MMP	Matrix metalloproteinase
MDR1	Multidrug resistance 1
MRP1	Multidrug resistance protein 1
PBS	Phosphate-buffered saline
BCA	Bicinchoninic acid
RIPA	Radioimmunoprecipitation assay
PAGE	Polyacrylamide gel electrophoresis
PVDF	Polyvinylidene fluoride
BSA	Bovine serum albumin
ECL	Enhanced chemiluminescence
SD	Standard deviation
