# Supplementary figures and images for: Polydopamine-Coated Surfaces Promote Adhesion, Migration, Proliferation, Chemoresistance, Stemness, and Epithelial–Mesenchymal Transition of Human Prostate Cancer Cell Lines In Vitro via Integrin α2β1–FAK–JNK Signaling
Source: Int J Mol Sci. 2026 Jan 8;27(2):655. doi: 10.3390/ijms27020655 (PMC12841494; doi:10.3390/ijms27020655)

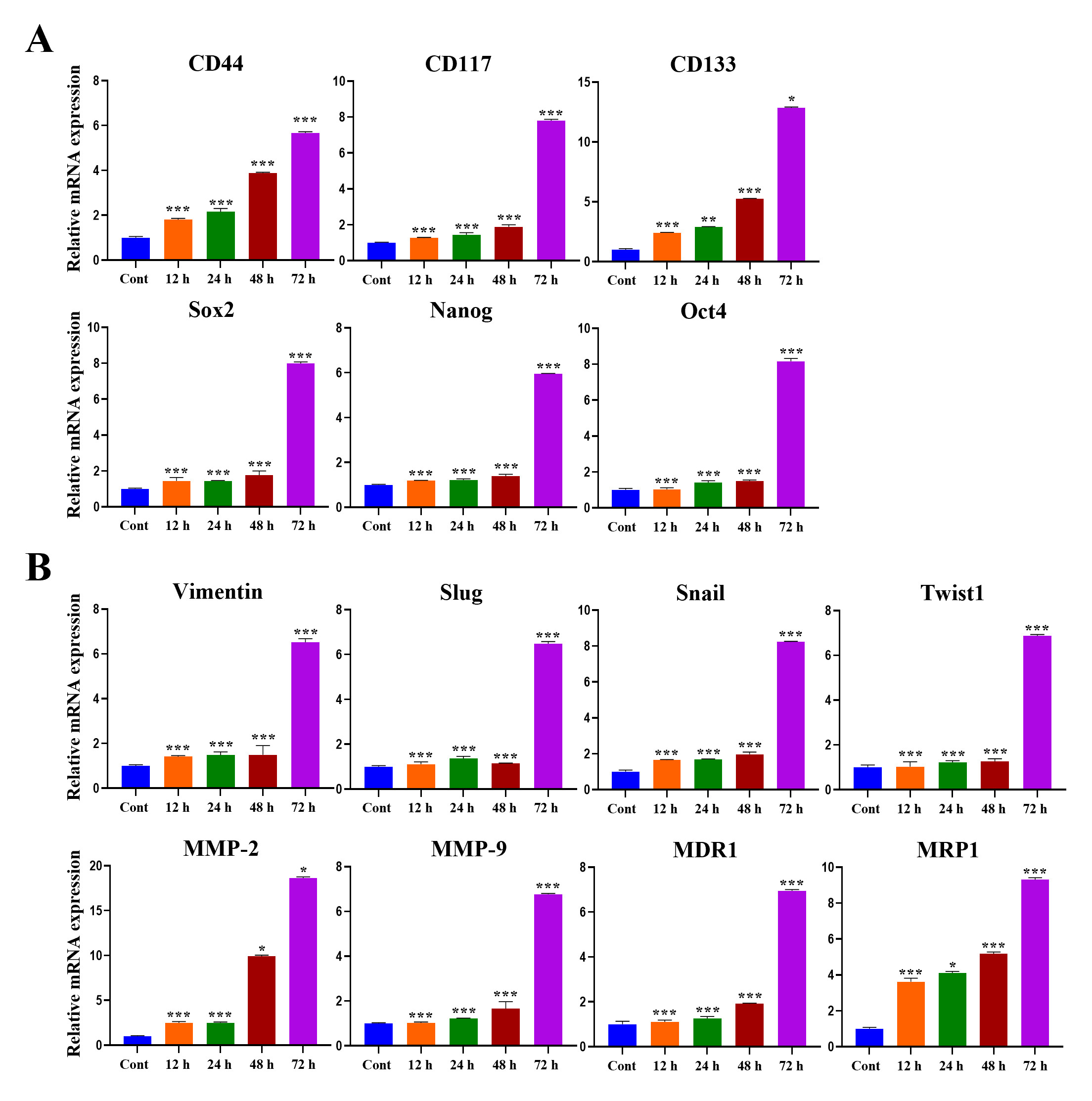

Supplement: Supplementary file 1 [file ijms-27-00655-s001.zip › ijms-4046285-supplementary.jpg]
